# Hybrid TiO_2_ Particles/Fluorinated Polymer as a Protective Layer for α-HgS Cinnabar: A Multi-Analytic Study

**DOI:** 10.3390/molecules31142429

**Published:** 2026-07-10

**Authors:** Federica Valentini, Pasquino Pallecchi, Irene Angela Colasanti, Camilla Zaratti, Andrea Macchia, Michela Relucenti, Loredana Cristiano, Nicoletta Volante, Ilaria Fratoddi, Sara Cerra

**Affiliations:** 1 Department of Sciences and Chemical Technologies, Tor Vergata University of Rome, Via della Ricerca Scientifica 1, 00133 Rome, Italy; ireneangela.colasanti@students.uniroma2.eu (I.A.C.); camilla.zaratti@students.uniroma2.eu (C.Z.); 2 Museo e Istituto Fiorentino di Preistoria, Via dell’Oriolo 24, 50122 Firenze, Italy; pasquino.pallecchi@unifi.it; 3 YOCOCU APS, Via Torquato Tasso 108, 00185 Rome, Italy; aps@yococu.com; 4 Anatomical, Histological, Forensic and Orthopaedic Sciences Department, Sapienza University of Rome, Via Alfonso Borelli 50, 00161 Rome, Italy; michela.relucenti@uniroma1.it (M.R.); loredana.cristaino@uniroma1.it (L.C.); 5 Historical Sciences and Cultural Heritage Department, Siena University, Via Roma 56, 53100 Siena, Italy; nicoletta.volante@unisi.it; 6 Department of Chemistry, Faculty of Science MFN, Sapienza University of Rome, P.le Aldo Moro 5, 00185 Rome, Italy; ilaria.fratoddi@uniroma1.it (I.F.); sara.cerra@uniroma1.it (S.C.)

**Keywords:** hybrid materials, fluorinated polymers, functionalized TiO_2_ particles, α HgS-cinnabar, egg yolk and wax/oil binders, multi-analytical approach

## Abstract

In recent years, hybrid materials have been widely applied in the cultural heritage conservation field, especially to preserve color pigments. Among these, one of the most problematic (in terms of conservation science) is the red pigment cinnabar/vermilion. The challenge of this work was to prepare a hybrid coating consisting of a fluorinated polymer (known to protect cinnabar/vermilion), further modified with an inorganic filler based on anatase TiO_2_. The latter is suitable because it is functionalized with quenchers, the particles are well above the nanoscale (≥200 nm in diameter), and it was added to the polymer matrix in small quantities. These characteristics made it suitable as a hybrid coating for protecting natural cinnabar, as demonstrated by the results obtained through a multi-analytical approach, based on multispectral imaging, Fourier-transform infrared spectroscopy (FTIR), scanning electron microscopy (SEM) coupled with energy-dispersive X-ray analysis (EDX), X-ray diffraction (XRD), X-ray photoelectron spectroscopy (XPS), X-ray fluorescence (XRF), contact angle, spectrophotometry and mechanical tests, which were applied to evaluate the performances of the hybrid coating on laboratory specimens (after aging) and original samples. The experimental results provide insight into both the physicochemical decomposition mechanism of natural cinnabar under laboratory-simulated aging conditions and the benefits of the coating. In particular, the treatment did not induce electrochemical changes in the mercury, which remained in its oxidized state (+2) rather than being further reduced to elemental mercury (Hg^0^), the species responsible for the blackening of cinnabar/vermilion (also combined with meta-cinnabar). In the oxidized form (Hg^2+^), the protein binder was altered, yet the application of the hybrid coating did not cause further physicochemical changes (i.e., red shift) to the Hg^2+^/egg-based binder system. This was also reflected in the color properties, which underwent no significant alteration. Finally, the mechanical tests yielded satisfactory results, particularly regarding water vapor permeability and treatment efficiency (even eight months after the initial application, although studies on the same samples are still ongoing). The hybrid coating was ultimately applied to original samples collected at Poggio Spaccasasso (Tuscany, Italy), which could be representative of prehistoric artworks based on natural cinnabar and traces of prehistoric adhesives made from beeswax, natural oils, and plant resins.

## 1. Introduction

It is well known that mercury sulfide (HgS, mainly as natural cinnabar and synthetic vermillion) tends to undergo darkening over time, mainly as a consequence of prolonged exposure to light, humidity, temperature fluctuations and environmental pollutants. Over the years, several mechanisms have been proposed to explain the degradation pathways for cinnabar (the natural HgS mineral) and vermillion (a synthetic pigment used in artistic applications), which could be expected to undergo similar degradation processes. One of the earliest hypotheses suggested a structural transformation of mercury sulfide into its black allotrope, metacinnabar, a thermodynamically less stable phase at room temperature. Although plausible under specific environmental conditions, this hypothesis alone is now considered insufficient to explain the phenomenon [1,2,3]. Subsequent studies highlighted the role of atmospheric pollutants, particularly sulfur dioxide (SO_2_). In the presence of moisture and calcareous substrates, SO_2_ can promote the formation of secondary products such as gypsum, thereby altering the pictorial surface and indirectly contributing to the blackening [3]. Furthermore, the interaction between sulfur compounds and the pigment can lead to the formation of mercury sulfates, such as HgSO_4_·H_2_O and schuetteite (Hg_3_(SO_4_)O_2_), which represent additional degradation products [4]. Another important mechanism involves the presence of chlorides, especially sodium chloride, which can react with the original pigment, leading to the formation of mercury chlorides such as calomel (Hg_2_Cl_2_) and corderoite (Hg_3_S_2_Cl_2_). The latter is a photosensitive and light-unstable compound that degrades further upon illumination, generating elemental mercury (Hg^0^) and other species, which significantly contribute to the darkening of the surface [2,4,5,6,7]. More recent research has demonstrated that blackening can also result from the formation of a colloidal phase of elemental mercury, produced through photo-induced redox reactions. In this context, the presence of salts, particularly chlorides, not only supplies reactive ions but also catalyzes processes, lowering the activation energy required for HgS reduction and thereby accelerating the formation of metallic mercury [1,4]. According to this, Anaf et al. (2013) [8] reported on several electrochemical experiments that confirmed the photodarkening of red vermilion (α-HgS) pigment in artworks, provoked by the formation of metallic mercury (Hg^0^), accelerated by light and chloride ions. Furthermore, by analyzing α-HgS|Pt electrodes, Anaf et al. (2013) [8] identified an oxidation peak at around −50 mV (representing the Hg^0^/Hg^2+^ oxidation) only after exposure to both light and chloride-rich environments. Other authors, such as Radepont et al. (2013) [9], demonstrated that the blackening of red mercury sulfide (α-HgS, cinnabar/vermilion) in artworks is not primarily due to phase transformation into black metacinnabar, but rather its photochemical degradation into metallic mercury and various mercury chlorides, induced by light, chlorine, and humidity. Radepont et al. (2013) [9] suggested that the degradation pathway seems to occur when light exposure facilitates the transformation of cinnabar into black, silver-grey, or white degradation products, mainly Hg chlorides (e.g., calomel Hg_2_Cl_2_ and polymorphs of Hg_3_S_2_Cl_2_) and metallic mercury (Hg^0^). These authors explained that the main factors are the presence of chlorine (from environmental sources like salts), light and humidity, which play a crucial role in the overall reactions. Radepont et al. (2013) [9] employed Pourbaix diagrams to confirm that the observed transformation products are consistent with electrochemical degradation in the presence of chloride ions and light. Artificially produced pigment tends to be less chemically stable than naturally extracted pigment. This is due to millennia-old geological processes that form compact and highly pure trigonal crystals (α-HgS) [10], compared with laboratory synthesis of vermilion, conducted by the wet or dry process with sulfur and mercury. This last procedure provides vermilion, a much more chemically vulnerable HgS-based compound. Historical production processes often lead to the formation of much more compact and highly pure α-HgS crystals (which explains the greater chemical stability of natural cinnabar compared with the synthetic vermilion) [10].

It should also be emphasized that one of the main causes of mercury sulfide degradation lies in the progressive deterioration of the binding medium used for pigment application or beeswax, animal fats (such as ruminant fat), and plant resins (such as pine pitch), widely applied as archaeological adhesives to fix the mineral pigment on stone tools (this last one being applied for hunting) and cave paintings [11,12]. The presence of tools and extracted cinnabar at the Spaccasasso archaeological area highlights the main role of the site as a vital resource hub, transitioning into a cemetery area during the Late Copper Age and Early Bronze Age, where cinnabar was heavily utilized in ancient ritual and burial practices [13]. Neolithic cinnabar tools and paintings, revealed at Spaccasasso, help contextualize the expansive ancient trade networks of the Italian peninsula, with the Tuscan cinnabar being transported to early settlements like La Marmotta in Lazio [14]. Recent archaeometric analyses of artefacts from the La Marmotta site (located on Lake Bracciano, Lazio) provide the earliest evidence of cinnabar (HgS) use in the western Mediterranean, dating back roughly 7500 years. This bright red pigment was deliberately chosen over local ochre for burial rituals, for body decoration, and on various tools [14]. The cinnabar found at La Marmotta was likely sourced from ore deposits in southern Tuscany, which are roughly 300 km away. The transportation and exchange of this high-value mineral highlight the sophistication of early Neolithic societies [14]. When the binder deteriorates, cinnabar becomes directly exposed to external factors such as light, humidity, and temperature variations. This loss of protection allows chemical and photochemical reactions to take place, ultimately leading to the pigment’s degradation [1].

In light of these considerations, the development of an effective protective treatment capable of restoring a barrier against environmental agents is of primary importance. However, conventional conservation methods, such as laser or plasma cleaning, as well as traditional conservation materials (e.g., polymers and gels), can alter the original raw materials by inducing local heating, color variation, mechanical stress, or even partial or total waterproofing of painted surfaces, thus disturbing the natural thermodynamic balance with the environment [15]. In the last two decades, nanomaterials (NMs), including nanoparticles (NPs), nanocomposites (NCs), nano-colloids (Ncs), nano-emulsions (NEs), and nano-dispersions (NDs), have shown great potential in the field of conservation, restoration, and cleaning of historical and artistic surfaces [16,17,18,19]. Their exceptional chemical–physical, biochemical, and mechanical properties at the nanoscale make them eco-sustainable [20], compatible with polychrome materials, biocompatible for end users, and highly versatile toward restoration of historical surfaces.

Nanomaterials are considered eco-sustainable [20] because their interesting features at the nanoscale—typically 1 to 100 nanometers—enable highly efficient, low-waste, and environmentally friendly solutions. By manipulating matter at this level, engineered materials show superior performance compared with their bulk, minimizing the need for raw materials and energy, while providing superior environmental remediation capabilities. Nanomaterials are transforming cultural heritage conservation by replacing toxic, petroleum-based solvents with eco-friendly, highly targeted alternatives. These “green” nanoscale systems are safer for both restorers and artifacts, offering superior penetration, structural compatibility, and long-term preventive protection for fragile paintings, stones, and historical manuscripts [21].

These features allow them to address a wide range of issues, from consolidation and surface protection to cleaning and re-adhesion of historical layers [22,23]. One of their most relevant properties, their high specific surface area [23], allows minimal quantities of nanomaterial to be used, ensuring non-invasiveness and full respect for the integrity of artwork objects. Another important feature is their tunable surface chemistry, which can be modified through functionalization with specific and selective groups. This approach has been widely explored for various applications, such as conservation and restoration treatments [15], drug delivery [24], environmental remediation [25], electrochemical sensing [26], and medical diagnostics and theranostics [27,28]. Recent studies have further clarified the mechanisms and potential of such surface modifications [29,30,31,32,33,34].

Among the nanomaterials recently applied in the cultural heritage field, SiO_2_ nanoparticles (SiO_2_ NPs) have proven particularly effective for the consolidation of Pietraforte stone materials [35] in Florence (Italy), the improvement of water-repellent efficiency of treated historical surfaces [36], and as self-cleaning coatings, especially when functionalized with organic natural molecules exhibiting antibacterial and antimicrobial properties [37,38]. In the conservation of ancient polychrome surfaces, particularly those containing the highly unstable mercury sulfide compounds [39], SiO_2_ NPs have already been successfully applied, as reported in the recent literature [40]. Afifi et al. (2023) [39] described their use in two formulations: an inorganic dispersion in 2-propanol and a hybrid form dispersed in hydroxypropyl cellulose (HPC, Klucel-E). The inorganic dispersion in 2-propanol does not induce color alteration and, owing to the solvent’s slow evaporation rate, allows the nanoparticles more time to penetrate the paint layer, improving the consolidation efficiency [37,41]. Conversely, the hybrid SiO_2_/HPC formulation provides higher solubility in water and a wider range of compatible solvents, lower adhesive strength, and no color change, thus preventing darkening effects on the painted surface [42,43,44]. Recent studies [40] also reported promising results for aqueous colloidal dispersions of SiO_2_ NPs used as binders in chromatic reintegration of wall paintings, with gloss values slightly lower than traditional fresco techniques but maintaining comparable wettability properties. More recently, Abdrabbo et al. (2025) [45] demonstrated that TiO_2_ nanoparticles, well dispersed in Klucel-E, could be successfully applied to cinnabar-based surfaces that were a part of a painted wooden canopic chest that dated between the late 27th dynasty (525 BC-404 BC) to the Greco-Roman period and is stored now at the storage rooms of the Grand Egyptian Museum in Giza (Egypt), showing good penetration and minimal color change. However, when using the inorganic TiO_2_ NPs alone, a higher degree of color alteration was observed, though consolidation performance remained satisfactory.

According to this, for the first time, this study focused on the new application of a TiO_2_/Fluoline CP hybrid material (referred to as TiO_2_ Ps/FCP for the rest of this manuscript, where Ps are particles) on laboratory specimens and the original sample (the latter collected in Poggio Spaccasasso, the Tuscany region, Italy). A two-fold study was conducted on artificially aged laboratory samples: first, to understand the physicochemical mechanisms of damage affecting cinnabar, and second, to evaluate the effectiveness of a hybrid coating treatment. Following the assessment of the hybrid coating’s satisfactory performance, it was applied to the original sample from Poggio Spaccasasso. This latter step serves as a preliminary study for applying conservation science to significant prehistoric artworks—such as rock paintings, hunting tools, and funerary objects—composed of natural red cinnabar and prehistoric adhesives made from beeswax, natural oils, and plant resins.

## 2. Results

This paragraph presents the characterization results of the laboratory samples (as-prepared and aged samples), before and after the conservation treatments, respectively. Only after having demonstrated and identified the best efficiency of the treatment (based on the hybrid material) was it then applied to the original sample selected for this study (Section 2.4).

The first characterization of the new hybrid material concerned the inorganic filler. Briefly, anatase TiO_2_ Ps showed a diameter of 200 nm, a surface area of 13.3 m^2^/g, a pore volume of 0.046 cm^3^/g and a pore size of 180 nm (as reported in Scheme S1, Supplementary Materials). FTIR analysis (Scheme S2, Supplementary Materials) showed the presence of some quenchers and scavengers, especially represented by the alcoholic functional groups on TiO_2_ Ps’ surfaces, which could have contributed to reducing the overall photo-efficiency (in combination with the minimum concentration of TiO_2_ Ps adopted in the formulation of the FCP composite materials).

### 2.1. Laboratory Specimens Before Treatments

**Figure 1:**
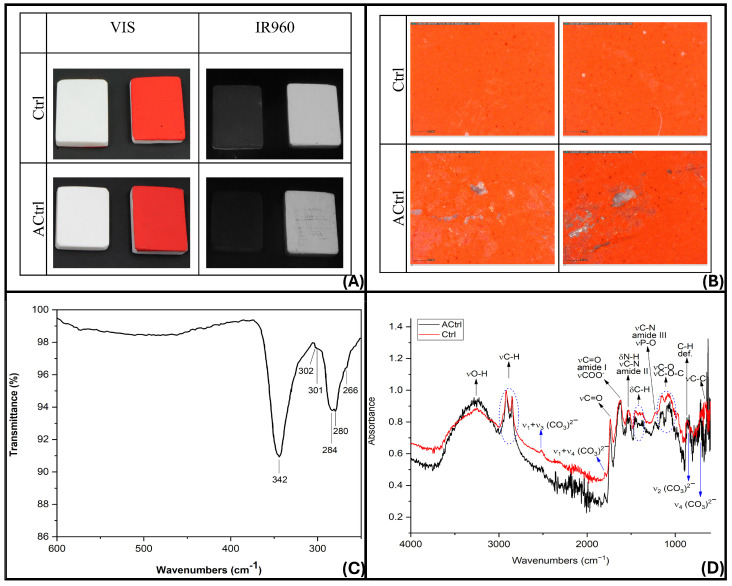
(**A**) Multispectral imaging (cinnabar samples were rotated to have marble) and (**B**) Dinolite analyses; (**C**) far-IR spectrum; (**D**) FTIR spectra for Ctrl and A Ctrl samples.

Multispectral imaging analyses performed on the samples before (Ctrl) and after artificial aging (A Ctrl) revealed the formation of a black patina, clearly visible both in the images acquired in the visible range (VIS) and, more evidently, in infrared reflectography (IR960) (Figure 1A). This chromatic alteration involved the pigment and is characteristic of cinnabar, which is known to undergo darkening phenomena under unfavorable environmental conditions.

The high-magnification observation of the surface (Figure 1B) highlighted the presence of fine lines and grey reflective areas (especially in the aged samples). The linear distribution of these features may be related to the pigment application method; indeed, residual brushstroke patterns were still partially visible on the samples, and thinner or more exposed areas may have undergone more pronounced degradation phenomena. The latter seemed to be associated with the formation of grey colloidal mercury compounds, especially in A Ctrl, probably related to the presence of oxidized Hg^2+^ forms (i.e., HgSO_4_ with the typical oxygen peak, β-HgS-metacinnabar; HgCl_2_ and Hg_2_Cl_2_, according to the XPS analysis, highlighted in Table 1). No Hg^0^ metallic components were detected by XPS (i.e., Hg^0^ was recorded at 99.7–99.9 eV). Meanwhile, the presence of Cl in the A Ctrl sample could be related to the photodegradation of α-HgS; the one found in the Ctrl could probably be due to the impurities, such as Hg_3_S_2_Cl_2_ (corderoite), contained in Kremer Pigments [1].

The presence of selenium/Se in RC is a natural interference due to the substitution of Se for sulfur in the alpha cinnabar lattice. The EDX spectra (in Figure S3, Supplementary Materials [46]) confirmed the XPS data.

**Figure 2:**
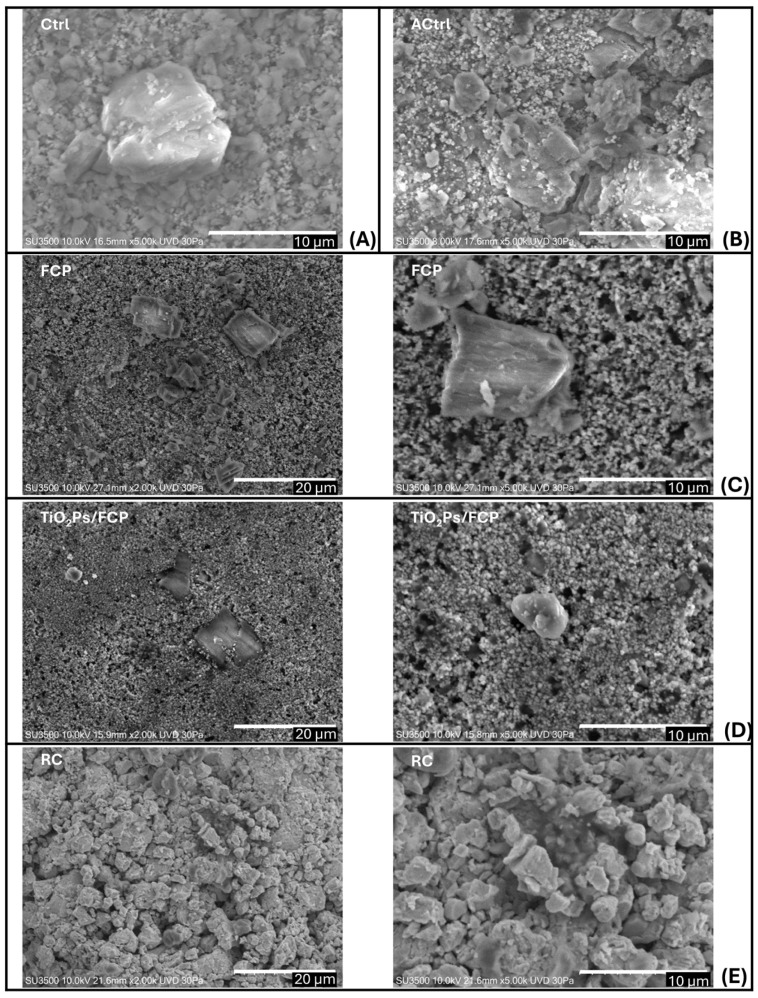
SEM micrographs of the investigated cinnabar samples: (**A**) Ctrl (unaged), (**B**) A Ctrl (aged), (**C**) FCP, (**D**) TiO_2_Ps/FCP, and (**E**) RC.

Figure 2A,B show the SE micrographs of the Ctrl and A Ctrl samples, respectively. At 5000× magnification, the Ctrl sample (Figure 2A) was characterized by tabular crystals of approximately 20 µm with smooth planar faces. Conversely, the aged A Ctrl sample (Figure 2B) exhibited smaller tabular to prismatic crystals, approximately 5–15 µm in size, with less defined edges and rougher surfaces, suggesting the occurrence of surface alteration. However, aging treatments did not induce the formation of Hg^0^ metallic drops in A Ctrl, according to the XPS analysis reported above.

Chromatic variation was further quantified by spectrocolorimetric analysis, which revealed an average ΔE* = 3.49 (Table 2). This value falls within the threshold of acceptability (3 < ΔE* < 5) but is still perceivable to the human eye. Specifically, the L* parameter remained almost unchanged, while both the a* and b* values decreased following artificial aging, resulting in a slight reduction in the red and yellow chromatic components. Considering that the reflective areas were small and localized, the color change remained significant, and it is reasonable to assume that more intense aging would have resulted in a more noticeable chromatic alteration.

To assess the degradation of the binder, contact angle measurements and FTIR spectroscopy were carried out. The contact angle, initially 98° ± 3° (hydrophobic condition), dropped to 50° ± 3° after aging (Table 2), indicating increased surface wettability. This is explained by the chemical degradation of the egg tempera binder, which undergoes cleavage of lipid chains with the release of carboxylic acids and protein denaturation, resulting in a more hydrophilic surface.

FTIR analysis revealed the main vibrational bands associated with the protein–lipid organic matrix of the binder (Figure 1D, Table S1 in Supplementary Materials). In the unaged sample, a broad band at 3257 cm^−1^ was observed, attributed to the stretching vibrations of νO–H and νN–H groups, together with bands between 2950 and 2850 cm^−1^ related to the C–H stretching of methyl and methylene groups. The lowest molecular band, centered at 2519 cm^−1^, is also attributable to calcium carbonate. A distinct band at 1739 cm^−1^ corresponds to the νC=O stretching of ester carbonyl groups from lipids, while the amide I, II and III bands, located at 1629, 1539 and 1227 cm^−1^ respectively, are assigned to νC=O, δN–H + νC–N and νC–N vibrations, the latter partially coupled with νP=O contributions from phospholipids or phosphoproteins. A 1795 cm^−1^, a lower-intensity band is attributable to calcite. The band centered at 1539 cm^−1^ hides the typical calcite band (attributable to the substrate) in the area between 1400 and 1450 cm^−1^. Furthermore, between 700 and 1000 cm^−1^, there are also the less-intense bands of calcite (713 and 876 cm^−1^), referred to as Carrara marble, applied for the laboratory samples’ preparation. The spectrum recorded at low frequencies (e.g., 225–600 cm^−1^) allows us to better identify the characteristic bands of HgS, all attributed in Figure 1C. The band at 301 cm^−1^ (typical of HgS) covers that small evident shoulder centered at 302 cm^−1^, which is attributable to calcite (Figure 1C) [47].

After artificial aging, the FTIR spectrum showed significant variations in both the position and intensity of the main functional bands, indicative of degradation processes affecting the organic component (i.e., cross-linking, hydrolysis, and oxidation). In particular, the decrease in intensity of the band at 3251 cm^−1^ reflected a reduction in hydrogen bonding, consistent with protein denaturation and matrix dehydration. Reductions alongside the 3251 cm^−1^ band also implied a decrease in the olefinic =CH stretching (around 3010 cm^−1^) [1,48,49,50]. The quoted observation perfectly describes photolytic oxidation and lipid degradation. Exposure to UV light breaks the double bonds in the cis-configuration (which gives the fatty acid chains their characteristic “kinked” shape), fundamentally altering the molecular structure of the egg yolk lipids over time.

The shift and increase in the carbonyl band from 1739 to 1735 cm^−1^ indicate the formation of new C=O groups due to lipid oxidation and the production of secondary carbonyl compounds such as aldehydes, ketones and esters. The appearance of a second band at 1711 cm^−1^ after aging is indicative of free fatty acids released after hydrolysis of glycerol esters [4] and references cited therein, which can further accelerate the oxidative processes of lipids. In the presence of cinnabar (Figure 1D), the intensity of the ester band decreased, and a band centered at 1705 cm^−1^, associated with free fatty acids, was observed after aging [51]. A shoulder centered at 1776 cm^−1^ (only detected in pure egg yolk [52]) in the presence of cinnabar appeared to be undetectable, and this was probably combined with the oxidative polymerization of fatty acids [4] and references cited therein.

The relative increase in the amide II band (1525 cm^−1^) compared with amide I (1623 cm^−1^) suggests conformational rearrangements and a transition towards β-sheet structures, typical of denatured proteins. In the presence of cinnabar, the amide I band suffered a rapid decrease in intensity, and the component at 1630 cm^−1^ shifted to 1623 cm^−1^, suggesting the presence of aggregated protein as a result of oxidation [4]. The amide II band at 1539 cm^−1^ in the presence of cinnabar decreased in intensity and showed a small red shift (after aging), probably linked to the formation of stable protein–metal complexes, involving amide groups and Hg^2+^ ions [4] and references cited therein. These important organic–inorganic interactions, which resulted in the oxidation of the organic binder, occurred more frequently when the one being applied was a finer-grained pigment (see Table 3).

The increased intensity of the bands at 1452, 1401, and 1374 cm^−1^ is attributed to the accumulation of aliphatic oxidative products, while the amide III band (~1220 cm^−1^) becomes more intense but less defined, suggesting a loss of structural order within the protein backbone due to denaturation. Furthermore, the enhanced absorption near 1150 cm^−1^ (νC–O) is consistent with lipid hydrolysis and oxidation, leading to the formation of alcohols, acids, and esters. Finally, the appearance of a new band at 659 cm^−1^ suggests the formation of oxidized secondary species, likely related to advanced degradation reactions of the binder or to interactions between the organic components and the pigment [1,48,49,50]. All the molecular band assignments are reported in Table S1 in the Supplementary Materials.

The same characterization measurements were performed on treated samples (by applying FCP and FCP hybrid polymers, before and after aging), and the results are summarized in Section 2.2 and Section 2.3, respectively.

### 2.2. Laboratory Specimens After FCP Treatments

**Figure 3:**
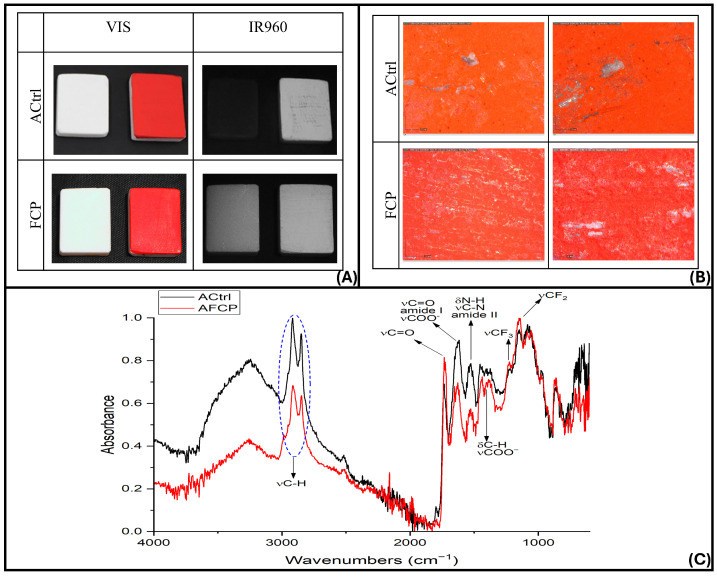
(**A**) Multispectral imaging and (**B**) Dinolite analysis; (**C**) FTIR spectra for FCP sample.

Multispectral imaging revealed a lower infrared reflectance, as indicated by the reduced contrast between the control sample (marble) and the painted area treated with Fluoline CP (Figure 3A). The presence of the coating also reduced the darkening effect, visible both in the VIS and IR reflectography images, confirming the formation of a superficial overlayer on the pictorial surface. This effect was particularly evident at higher magnifications (Figure 3B), where a decrease in the sub-metallic appearance of degraded areas and an increase in surface gloss could be observed after the application of the fluorinated polymer. However, no significant color variations were detected (ΔE* = 1.98), either in chroma (ΔC) or in hue (ΔH), as reported in Table 2. The formation of a continuous surface coating was further confirmed by the increase in the contact angle, which rose from 50° ± 3° for the aged sample to 106° ± 2.5° for the sample treated with Fluoline CP (Table 2).

SEM analysis of the artificially aged cinnabar-based pigment treated with Fluoline CP showed distinctive morphological features. At 2000× magnification (Figure 2C on the left), the sample displayed larger euhedral crystals (approximately 15–20 µm) having a well-defined tabular morphology. At 5000× magnification (Figure 2C on the right), the tabular crystal surfaces showed a smooth appearance with characteristic parallel surface features, suggesting a coating effect induced by the Fluoline CP treatment.

FTIR spectroscopy (Figure 3C) revealed the appearance of additional absorption bands and an increase in the intensity of specific signals associated with the coating. In particular, two new bands between 2980 and 2950 cm^−1^ were observed, corresponding to the asymmetric stretching of CH_3_ groups (ν_as_CH_3_) from the aliphatic chains of the polymer. Moreover, the increase in the absorption bands at 1730, 1230, and 1150 cm^−1^ is related to the C=O, –CF_3_, and –CF_2_ groups, respectively, which are characteristic of fluorinated acrylic resins [53]. The full band attribution is reported in Table S2 (Supplementary Materials), where the signal centered at 1730 cm^−1^, related to the ester groups, did not show a significant increase, indicating limited oxidative processes compared with A Ctrl, according to the literature [48]. As for the signals included in the intervals 1620–1623 cm^−1^ and 1539–1525 cm^−1^, slight variations (both in terms of wavenumber shift and less pronounced intensity increases) were observed when compared with A Ctrl, highlighting a protective effect of the FCP coating [53]. Furthermore, the C–H stretching vibrations at approximately 2980, 2950 and 2917 cm^−1^ remained essentially unchanged, indicating that no substantial alterations occurred within the organic matrix. A slight shift within the 1417–1401 cm^−1^ region was also detected, which may reflect a reduced contribution of oxidation-related species, consistent with the overall spectral features associated with the protective action of the coating [53,54,55]. In the low-frequency spectrum (225–800 cm^−1^), there were no characteristic signals for FCP, as it is a mixture of two components: fluoroelastomers and acrylic polymers dissolved in acetone (both amorphous without crystalline phases). This low-frequency spectrum (400–800 cm^−1^) would instead be important for fluorinated polymers (applied in the field of restoration), such as poly (vinylidene fluoride) (PVDF), which present three distinct crystalline phases, according to the literature [53].

Table 3 clearly shows that the contact angle significantly increased (i.e., 106° ± 2.5°, when compared with the 98° ± 3° value acquired in the presence of Ctrl), according to the waterproofing capacity of polymers.

### 2.3. Laboratory Specimens After TiO_2_ Ps/FCP Treatments

**Figure 4:**
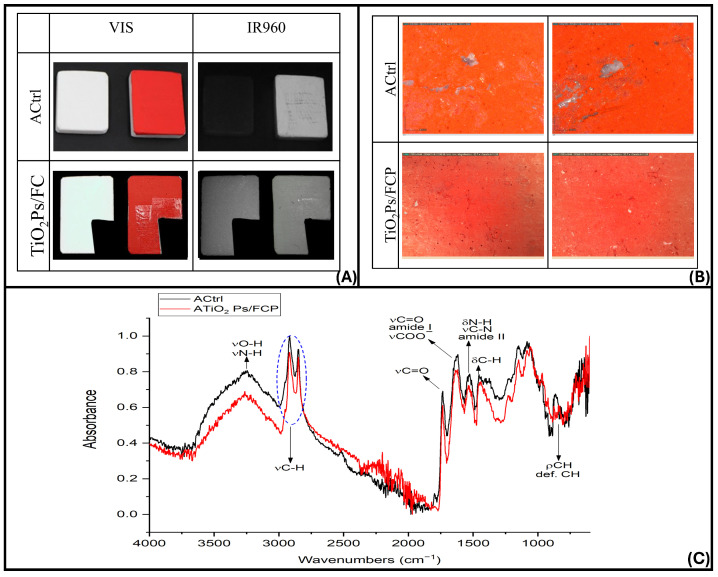
(**A**) Multispectral imaging and (**B**) Dinolite analyses; (**C**) FTIR spectrum for TiO_2_ Ps/FCP sample.

Multispectral imaging analysis (Figure 4A) showed the laboratory mock-up treated with the hybrid coating based on FCP/TiO_2_ Ps (on the left) and the control mock-up (the control was a marble sample where cinnabar had been placed, right). In the visible light, the treated sample retained its vivid red hue. In the infrared reflectography image (IR 960), the presence of the coating reduced the darkening effect visible in the aged control sample. Furthermore, the images revealed a similar reduction in infrared reflectance compared with the sample treated with FCP alone (Figure 4A). However, the treated surface exhibited an even more uniform appearance in both visible and infrared light. The red hue remained intense, while the typical darkening effect observed in aged samples was further attenuated. These observations confirmed the formation of a superficial protective layer, with enhanced optical modulation likely due to the presence of titanium dioxide particles, which increased light scattering and contributed to a more homogeneous visual aspect. This aspect was even more evident in the microscopic images (Figure 4B), where the sample treated with the hybrid coating showed a smoother and more coherent surface compared with the aged untreated control sample, and slightly more uniform compared with the surface treated with FCP alone (Figure 3B). The addition of TiO_2_ appeared to improve the film distribution, minimizing surface discontinuities. SEM analysis of the artificially aged cinnabar-based pigment treated with TiO_2_ Ps/FCP hybrid material revealed a distinctive morphology. At 2000× magnification (Figure 2D, on the left), the sample displayed a very fine and homogeneous matrix of submicrometric particles. Scattered larger crystals (approximately 15–25 µm) with tabular to elongated morphologies exhibited smooth surfaces and well-defined edges. At 5000× magnification (Figure 2D, on the right), particle aggregates with lobate morphology and smooth, uniform surfaces were observed, suggesting an effective coating by the treatment. The FTIR-ATR spectrum (Figure 4C) confirmed the presence of a hybrid fluorinated coating. The spectrum showed the characteristic absorption bands of the fluorinated polymer matrix, as previously discussed. A broad absorption feature in the 1000–950 cm^−1^ region, not evident in the FCP-only treated sample, is attributed to Ti–O–Ti stretching vibrations, confirming the integration of TiO_2_ particles within the coating [56]. The full band attribution is reported in Table S3, where the signals recorded in the range of 1730–1735 cm^−1^ (associated with the ester groups) highlight a weak but lower increase than that recorded in the presence of the Ctrl sample, indicating reduced oxidation processes. Meanwhile, the spectral range of 1530–1560 cm^−1^ shows a shift toward higher wavenumbers, suggesting a successful interaction between the polymer matrix and TiO_2_ Ps. In addition, the C–H stretching (2980–2850 cm^−1^) and bending (~1450 cm^−1^) vibrations remain substantially unchanged, indicating limited degradation phenomena and good stability of the protective system [53,54,55,56,57].

Table 2 shows that the contact angle (116° ± 2.0) exceeds the value acquired in the presence of the only polymer FCP (106° ± 2.5°), improving the waterproofing of the material surface and guaranteeing the correct permeability to environmental vapor. The colorimetric data (from Table 2) show an L* lower than that measured for the Ctrl sample, and this means that the TiO_2_ clusters had not yet reached a critical size to alter the macroscopic reflectance.

### 2.4. Original Samples: Characterization Study Before and After Treatments Based on TiO_2_ Ps/FCP

The original cinnabar sample was analyzed without any preliminary treatment.

**Figure 5:**
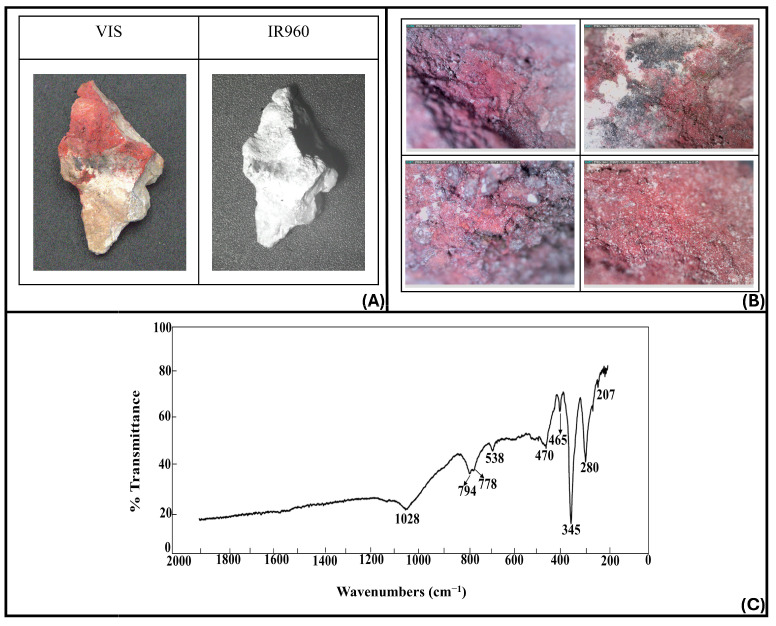
(**A**) Multispectral imaging and (**B**) Dinolite analyses; (**C**) FTIR spectrum for RC sample.

Figure 5 provides an overview of the analyses carried out.

Multispectral imaging revealed a moderate infrared reflectance with limited contrast between the cinnabar-painted area and the carbonaceous substrate in the IR reflectography image (Figure 5A, right). In visible light (Figure 5A, left), the pigment appeared as an intense red with uniform distribution.

Microscopic examination performed at different magnifications (Figure 5B) showed the presence of a coarse-grained surface morphology, typical of a natural pigment.

**Figure 6:**
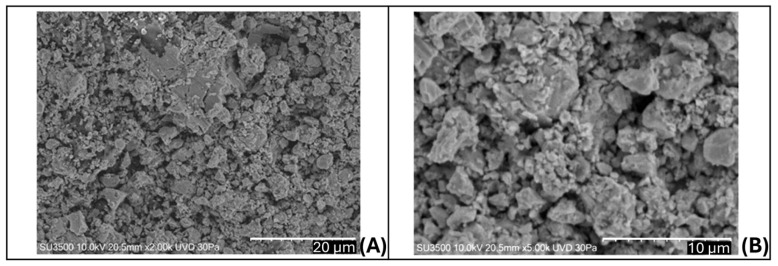
(**A**): SEM micrograph of RC treated with TiO_2_ Ps/FCP at 2000× magnification. Scale bar: 20 µm. (**B**): SEM micrograph of RC treated with TiO_2_ Ps/FCP at 5000× magnification. Scale bar: 10 µm.

SEM analysis of the cinnabar rock samples revealed a heterogeneous population of particles. At 2000× magnification (Figure 2E, left), crystals displayed subhedral to anhedral habits with particle sizes ranging from approximately 3 to 20 µm. Stubby prismatic to tabular morphologies were observed, along with fragmented particles showing irregular outlines. At 5000× magnification (Figure 2E, right), the crystal surfaces appeared smooth with sharp edges and step-like features. Subconchoidal fracture surfaces and adhering submicrometric particles were also observed. X-ray fluorescence (XRF) spectroscopy was subsequently employed to obtain a bulk quantitative assessment of the elemental composition of the sample (Figure S2 and Table S5). The XRF results confirmed that the sample was predominantly composed of Hg (≈60 atomic %) and S (≈24 atomic %), in agreement with the EDX observations. A significant Ca content (≈16 atomic %) was also detected, which was attributed to the presence of a carbonaceous and mineral matrix associated with the natural substrate of the sample. Far-infrared spectroscopy (FIR) highlighted the presence of cinnabar in the ore spectrum (Figure 5C), where the typical bands centered at 345 and 280 cm^−1^ were very well evident [58]. In the ore spectrum, there were also traces of silicate minerals (with bands recorded at 1028, 538 and 470 cm^−1^) and quartz (having a doublet centered at 794–778 cm^−1^, 465 and 207 cm^−1^). Furthermore, the cinnabar ore contained accompanying minerals of calcite (Figure S1), which appeared as white clumps or veins on the surface (see also Figure 6). The spectrum acquired in FTIR-ATR mode (Figure S1) also revealed the band centered around 1248 cm^−1^, attributable to the asymmetric stretching of SO_2_ (ν_as_) from α-HgS [49].

Morphological characterization after TiO_2_ Ps/FCP-based treatment, performed on RC samples, showed that at 2000× magnification (Figure 6A), larger crystals (approximately 15–25 µm) with tabular to prismatic habits exhibited smooth planar faces with well-defined edges and step-like surface features, surrounded by a heterogeneous population of smaller subhedral to anhedral particles. Aggregates of smaller particles with lobate to irregular morphology and relatively smooth, uniform surfaces were also observed (Figure 6B), suggesting the deposition of the TiO_2_ Ps/FCP coating on particle surfaces. The coating appeared to bridge between adjacent particles in some areas, partially filling interparticle spaces and conferring a smoother surface texture to the aggregates (and this supports the quality of the composite-based coating). Notably, no evidence of macroscopic TiO_2_ aggregation was observed at any magnification level; the fine particles attributable to the treatment appeared uniformly dispersed within the polymeric matrix and on particle surfaces, without forming discrete clusters or segregated domains (and this justifies the L* values, which did not increase, avoiding the possible whitening effect due to titanium dioxide).

The morphological characterization was also combined with the investigation of those mechanical parameters aimed at establishing the efficiency of the treatments, based on the hybrid polymeric materials. This study is summarized in Table 4, where quantitative information (concerning the surface area coverage, the total pore volume, the contact angle, etc.) is collected for before and after the TiO_2_ Ps/FCP-based treatment.

The colorimetric investigation justified the L* values, which did not increase, avoiding the possible whitening effect due to titanium dioxide (Ø = 200 nm).

In Table 5, the increment in superficial hardness (%), drilling resistance forces (DR/N), tensile strength (MPa) and treatment efficiency (%) are measured and reported.

To verify the significance of the ET% and P% data (acquired by measuring on the same identical specimens or areas of these samples), Student’s *t*-test for paired data was applied (considering that the difference between the permeability percentages at 0 and 8 months followed a normal distribution). Since the two-tailed *p*-value equaled 1.0000, by conventional criteria, these differences were considered to be not statistically significant (over the investigated time period between t = 0 and after t = 8 months). However, further studies should conduct tests on the same samples to verify, over longer time periods, the statistical significance of the differences in values measured for ET% and P%, respectively.

## 3. Discussion

The results obtained provide two important insights: the first clarifies the damage mechanism that α-HgS cinnabar undergoes after aging; the second evaluates the effectiveness of the new hybrid coating, better clarifying aspects such as the role of TiO_2_ Ps into the FCP polymeric films and the choice of RC sample.

XPS, SEM and FTIR were very useful in clarifying the chemical–physical mechanism of damage induced to cinnabar (after aging). The XPS results especially highlight the absence of metallic Hg^0^ and the presence of sulfate anions and chlorine/Cl species, confirming that the possible photodegradation mechanism of cinnabar could be represented as follows (Equations (1)–(4)):

(1) $$HgS+{4H}^{+}+{2H}_{2}O+O_{2}\to {Hg}^{2+}+{SO}_{4}^{2-}+{4H}^{+}$$

(2) $$2{HgSO}_{4}\left(aq\right)+{2e}^{-}↔{Hg}_{2}^{2+}+{SO}_{4}^{2-}is0.83Vvs.SHE(E^{0}=0.00\;V)$$

(3) $$2HgS+O_{2}+4{Cl}^{-}+{4H}^{+}\to {Hg}_{2}{Cl}_{2}+{4S}^{0}+{2H}_{2}O$$

(4) $$HgS+{2O}_{2}+2{Cl}^{-}\to Hg{Cl}_{2}+{SO}_{4}^{2-}$$

The photodegradation of natural cinnabar (α-HgS) in the presence of chlorides (e.g., Cl^−^ anions and/or molecular chlorine/Cl_2_) and in the absence of metallic mercury is an accelerated photo-corrosion process. This reaction provokes the well-known blackening of natural α-HgS, caused by the transformation of the red sulfide into secondary mercury compounds (e.g., β-cinnabar, calomel, hybrid corderorite and Hg-based sulfate secondary compounds). The SEM results never highlighted the presence of metallic Hg droplets on the surface of the aged samples (A Ctrl). This hypothesized Hg-based degradation mechanism completely differs from those recently reported in the literature [4], where Hg^0^ metal (in the absence of chlorine species, detected by XPS analysis) was deposited on cinnabar pigment-based painting surfaces (with evident Hg^0^ metallic droplets, acquired on cinnabar-based pigment micrographs), provoking darkening [4] and references cited therein.

The observed oxidation of S^−2^ led to Hg^2+^, which was able to chemically interact with organic binders, as the FTIR study showed. Especially, red shift and significant intensity signal modifications were recorded in the typical spectral fingerprint of proteins. This latter was clearly evident for the A Ctrl sample, where the amide II band recorded at 1539 cm^−1^ in the presence of cinnabar decreased in intensity and also showed a small red shift (after aging), probably linked to the formation of stable protein–metal complexes, involving amide groups and Hg^2+^ ions.

In the presence of a hybrid coating, what was observed was that in the FTIR, there were no additional red shift effects and/or alterations in the intensity of the molecular absorption bands in the amide’s region. The coating appeared homogeneous and uniform (as shown in the SEM micrographs), without areas of discontinuity, where the aggregated clusters of TiO_2_ particles were completely absent. This happened because the TiO_2_ Ps (having a 200 nm diameter and being present in small quantities in the formulation of the hybrid composite) were functionalized with alcohol groups (i.e., quenchers), with which they chemically bound to the FCP (see Scheme S1 in the Supplementary Materials). A homogeneous and uniform coating was guaranteed by TiO_2_ anatase particles, which promoted interfacial adhesion, acting as an anchoring bridge able to prevent the resin from “shrinking” in drops. Furthermore, by adding the TiO_2_ Ps as mineral filler, the wettability of the formulation (Fluoline FCP) on the substrate was adjusted, preventing the formation of accumulations (which could provide a whitening effect, typical of aggregated anatase clusters) and promoting a flat and continuous application. This was reflected in the L* parameter (which did not increase because there was no bleaching by the inorganic TiO_2_ filler, as mentioned above) but also in the other parameters measured in the color study (a* and b*, respectively), which were all lower than those measured for the A Ctrl sample (see Table 4). In summary, the coating toned down the warm tones, shifting towards softer shades, often approaching a more neutral or colorless appearance. The depth profiling XPS analysis also confirmed the absence of metallic Hg^0^ (after the hybrid-coating-based treatment) in the innermost region close to the substrate. The XPS signals detected on the surface (in the outermost layer) are typical of FCP (688.0 eV for CF_2_ bonds and 689.0 eV for CF_3_ bonds), combined with the C 1s region (CF_2_- recorded a 291.5 eV). In the intermediate/surface layer, the Ti 2p showed main peaks (Ti 2p_3/2_) at 458.6 eV and (Ti 2p_1/2_) ~464.3 eV, confirming the octahedral coordination of Ti^+4^ typical of anatase/TiO_2_. If the binding energy of the main peak (Ti 2p_3_/_2_) remains strictly confined to 458.5–458.7 eV and satellite peaks are absent, the presence of Ti^+3^ (which has lower binding energies of 457.0–457.5 eV) and “oxygen vacancies” (lattice defects in TiO_2_) is ruled out.

The hybrid coating performances were even better when applied to RC samples. This original sample was chosen because it was representative of archaeological prehistoric artworks, widely found in the Tuscany region (Italy), mainly in Poggio Spaccasasso and Monte Amiata. The link between Poggio Spaccasasso (in the Maremma Regional Park) and Monte Amiata is both geological and archaeological: they share an ancient and fascinating common thread connected to the extraction of cinnabar (the mineral from which mercury and the color red are derived). Numerous prehistoric archaeological artifacts have been found in these central Italian regions, all characterized by the presence of natural cinnabar, and—from the perspective of the conservation science of cultural heritage—are very little studied. Historical sources [59] shed light on archaeological adhesives (found in trace amounts and composed of beeswax, mineral oils, and plant resins (ref. [60]) used primarily to bond cinnabar to rock paintings, prehistoric stone hunting tools, and funerary artifacts (discovered in Tuscan necropolises). Therefore, in light of what has been described so far, initiating research into treatments for preserving natural cinnabar—both with and without organic binders (specifically those based on natural compounds rather than egg, such as those documented in archaeological and prehistoric sources [59])—would undoubtedly open new horizons in conservation science regarding cultural heritage rich in information on the past technologies, uses, and traditions of prehistoric civilizations.

The results obtained from the morphological analysis after RC treatment demonstrate that, despite the heterogeneous starting material, evidence of effective coating deposition was observed. Particle aggregates with lobate morphology and smooth, uniform surfaces were present, analogous to those documented in the TiO_2_ Ps/FCP-treated laboratory specimens (see above, in the full text). This suggests that the treatment mechanism is consistent regardless of the degree of prior processing of the mineral. The hybrid coating appeared to partially fill interparticle spaces and bridge adjacent particles, contributing to aggregate formation and supporting the quality of the composite-based coating. The smooth surface texture of these aggregates contrasts with the rough, angular surfaces of untreated raw cinnabar particles, supporting the formation of a covering layer by the TiO_2_ Ps/FCP formulation. Of particular significance is the absence of macroscopic TiO_2_ aggregation within the polymeric body of the formulation: at all magnification levels examined (1000–5000×), no discrete TiO_2_ clusters or segregated domains were detected, indicating a homogeneous dispersion of the photocatalytic component within the FCP matrix. This observation is critical, as the aggregation of anatase-phase TiO_2_ would result in localized concentrations of photocatalytic activity, potentially inducing degradation of both the polymeric binder and the underlying pigment substrate. These latter morphological findings support the colorimetric data (specifically L*, a*, and b*, which did not show increasing values following the application of the hybrid coating). Furthermore, the absence of metallic Hg^0^ droplets (as evidenced by SEM micrographs on RC) is consistent with the XPS results, where the Hg chemical shift is characteristic of alpha-cinnabar (see Table 2 for the substrate), and depth profiling analysis revealed signals at the surface (in the outermost layer) typical of FCP (688.0 eV for CF_2_ bonds and 689.0 eV for CF_3_ bonds) and C 1s region (CF_2_- recorded a value of 291.5 eV). In the intermediate/surface layer, the Ti 2p region exhibited the main peaks (Ti 2p_3/2_) at 458.6 eV and (Ti 2p_1/2_) ~464.3 eV, confirming the octahedral coordination of Ti^4+^ typical of TiO_2_ (anatase phase, as also indicated by XRD analysis in the Supplementary Materials).

RC TiO_2_ Ps/FCP showed a smaller surface area as well as a smaller total pore volume, and this suggests that the coating was well deposited on the cinnabar. The contact angle increased, demonstrating the reduced wettability of the cinnabar surface after treatment with the hybrid composite. An increase in tensile strength in a nanocomposite coating means that the dispersion of the TiO_2_ fillers and interaction with the polymer matrix have occurred optimally, creating a structure that better resists mechanical stress.

Despite the reduced wettability of the treated RC surface, water vapor permeability remained constant even eight months after the initial application (although measurements on the same samples are still ongoing to evaluate P (%) performance over longer periods). The results obtained contribute to a satisfactory treatment efficiency (ET%), particularly regarding the consistency of the measured values even after eight months (and, for this parameter as well, measurements are continuing on the same samples for long-term evaluation). Student’s *t*-test showed that there were no statistically significant differences between these two quantities measured at the time of the first application and after 8 months (from the first application). Studies are still underway on the same samples to evaluate permeability and efficiency over much longer time periods (which are more realistic in the case of the original samples under study).

The validity and statistical significance of these data, also collected on RC, open the door to an important study of prehistoric cinnabar samples, which are numerous but still too little studied for the purposes of preserving prehistoric archaeological cultural heritage.

## 4. Materials and Methods

### 4.1. Sample Preparation

Laboratory specimens: Laboratory samples were prepared on Carrara marble blocks (5 × 3.5 × 1 cm^3^ rectangular parallelepiped). The pigment cinnabar was applied using the tempera technique. A fresh egg was used. The egg white was discarded, and only the yolk was retained. The outer membrane of the yolk was removed, and the inner liquid portion was collected. An equal volume of distilled water was added to the yolk, and the mixture was manually beaten to initiate the natural denaturation of egg proteins, forming the tempera binder. This binder was gradually mixed with cinnabar powder (Red Cinnabar # 10620 produced by Kremer Pigments Inc., Aichstetten, Germany; Table 3 [61]) until a smooth and homogeneous paste was obtained, suitable for application onto the marble specimens. The painted samples were then left to dry under a laboratory fume hood at ambient conditions for 24 h. The coding of the all the prepared samples is reported in Table 6.

**Figure 7:**
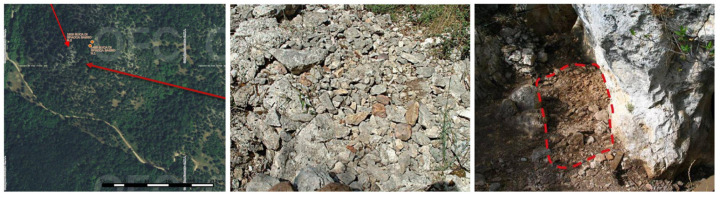
Sampling area on Poggio Spaccasasso mineralization (Tuscany region, Italy). The red arrows indicate the sampling location, while the dashed red outline identifies the specific area from which the sample was collected.

**Figure 8:**
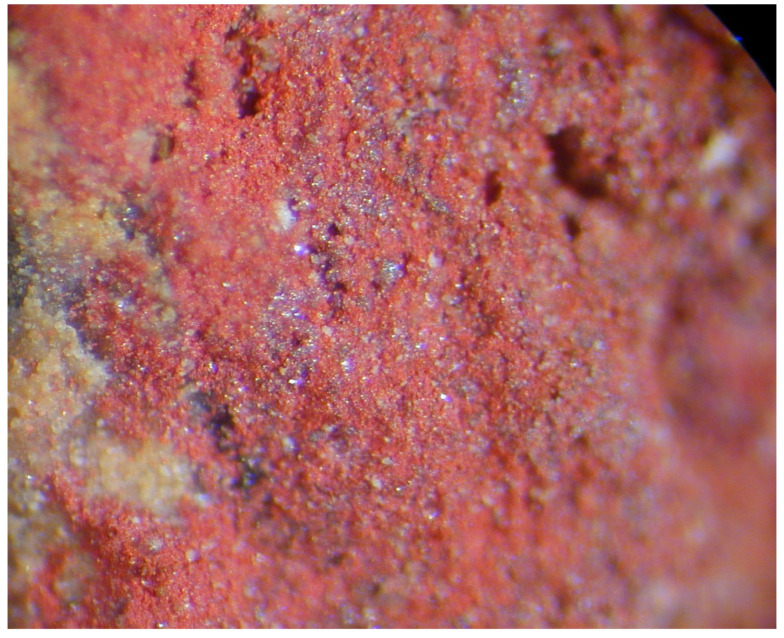
Details of the cinnabar encrustation before removal from the calcareous substrate.

Original cinnabar sample: The cinnabar sample came from a modest mineralization located in the Poggio Spaccasasso area. The mineralization affects fractures and karst cavities of the Jurassic limestone formation (Calcare Massiccio) that makes up the northern part of the Uccellina Mountains ridge on the coast south of Grosseto (Tuscany) (Figure 7). Cinnabar occurs in isolated encrustations and thin coatings on limestone surfaces together with traces of iron oxides and hydroxides (hematite and goethite) and can be traced back to the Plio-Quaternary hydrothermal activity that affected southern Tuscany. The mineralization was recently discovered during the exploration of a cavity in the limestone formation affected by the presence of numerous archaeological finds, including tools used for the extraction of cinnabar dating back to the Neolithic period to produce red pigment [13]. The analyzed sample (Figure 8) consisted of a thin granular encrustation of cinnabar separated from the calcareous substrate on which it was originally found.

### 4.2. TiO_2_ Ps/FCP Chemical Synthesis

Pristine TiO_2_ Ps were synthesized following the protocol reported in our previous study [56]. Briefly, Ps were obtained by adding 5 mL of titanium isopropoxide (used here as a precursor; Sigma-Aldrich Chemie GmbH, Taufkirchen, Germany) to 5 mL of absolute ethanol (Sigma-Aldrich Chemie GmbH, Taufkirchen, Germany) in an acidic working medium (i.e., H_3_PO_4_), and then the reaction mixture was stirred at room temperature (RT) for 3 h. This mixture was subjected to sol–gel hydrolysis of titanium (IV) isopropoxide (TTIP), and the resulting solid powder was filtered, thoroughly washed (by bi-distilled water; Milli-Q^®^ IQ Water Purification System; Merck KGaA, Darmstadt, Germany) and dried at 60 °C in an oven. In Scheme S1 (in the Supplementary Materials), a detailed description of the TiO_2_ Ps’ synthesis is also reported. TiO_2_ particles (as an inorganic filler) were dispersed in the Fluoline CP polymer matrix (commercially available from CTS Altavilla Vicentina (VI), Italy), in a ratio of 0.2% *w*/*w*, and the resulting hybrid compound (TiO_2_ Ps/FCP) was treated with a Hielscher Ultrasonics UP200Ht polytronic probe (Hielscher Ultrasonics GmbH, Teltow, Germany), enabling the dispersion of the solid in the polymer medium for at least an hour. The optimized concentration of 0.2% *w*/*w* was selected because previous studies have shown that particle loadings below 1% provide an appropriate balance between improved functional performance and the preservation of the aesthetic appearance of the treated surfaces, whereas higher concentrations tend to induce visible color variations and undesirable optical changes [62]. Reducing the concentration of TiO_2_ particles in the fluoropolymer formulation (lower nanoparticle concentrations) helps suppress overall photo-efficiency [63].

### 4.3. Artificial Aging Processes

After evaluating the initial conditions of the laboratory samples immediately following the application of the paint layer, the specimens were subjected to artificial aging in order to simulate the natural degradation and blackening processes typically affecting the cinnabar pigment. The protocol consisted of two weeks in a climate chamber (ACS Angelantoni Climatic Systems, Ofterdingen, Germany), working at a temperature ranging from 48 to 60 °C and at a relative humidity in the range of 40–60%. Subsequently, the samples were subjected to controlled UV irradiation inside an Aura Mini fume hood (BioAir, Siziano, Italy), equipped with a EuroClone UV lamp (model: AK30100, 15 W; EuroClone S.p.A., Pero, Italy), for a period of two weeks. During this last step, the samples were not subjected to further humidity and temperature control, as reported in the literature [64].

### 4.4. Characterization Techniques

The samples were comprehensively characterized using complementary analytical techniques to investigate their morphological, structural, chemical, optical, and surface properties before and after artificial aging, as well as to evaluate the effectiveness of the applied protective treatments. The experimental conditions adopted for each analytical technique are described in the following sections.

#### 4.4.1. Optical Microscopy

Optical microscopy was employed to examine the morphological characteristics of the painted surfaces, compare non-aged and aged areas, and assess the properties of the protective films. Analyses were performed using a DinoLite AM411-FVW digital microscope (AnMo Electronics Corporation, Taipei, Taiwan), operating at magnifications from 50× to 200× under visible (VIS) illumination.

#### 4.4.2. Multispectral Imaging

Multispectral imaging was employed to systematically document the conditions of the samples and their constituent materials, assess degradation phenomena, and evaluate variations following the protective treatment. The samples were analyzed before and after artificial aging and after coating application, using different wavelengths for the following imaging techniques: visible radiation (VIS), ultraviolet fluorescence (UVF), and infrared reflectography (IR960). Imaging was performed with a CANON EOS M50 full-spectrum camera equipped with an EF-M 18–150 mm f/3.5–6.3 IS STM lens (Canon Inc., Tokyo, Japan). Ultraviolet fluorescence was captured using a HOYA UV-IR filter (Hoya Corporation, Tokyo, Japan) in combination with a B+W Yellow 495 F-PRO MRC 022 filter (Schneider Kreuznach, Bad Kreuznach, Germany) to eliminate the blue component of the UV lamp (UVF-G). Infrared reflectography images were obtained using a high-pass filter at 960 nm. The light sources included a 365 nm UV source filtered at 400 nm and a 950 nm IR illuminator. All images were white-balanced with a standardized color-check reference target to ensure comparability across the imaging modalities.

#### 4.4.3. XPS

X-ray photoelectron spectroscopy, providing the quantitative elemental composition and oxidation state of Hg and S of unaltered/aged cinnabar-based mock-ups and original samples, was performed by applying an Axis Ultra-DLD (Kratos Analytical Ltd., Manchester, UK) apparatus, using monochromatic Al Kα radiation. The equipment settings are reported here: the survey spectra were acquired at a 75 W X-ray source power and a 160 eV pass energy, and the high-resolution spectra of C, Hg, S, and Cl elements were acquired at a 225 W source power and a 20 eV pass energy, with a 1.33 × 10^−8^ Pa vacuum. The analyzed area was ~300 × 700 μm in size. Qualitative analysis and elaboration of the abundance percentage of sulfur and mercury species were performed using the CasaXPS software, version 2.3.24 (Casa Software Ltd., Teignmouth, UK). Hg and S binding energies were determined by using the C 1s transition at 284.6 eV, as a reference. The positions of the multiple doublets S 2p_3/2_–S 2p_1/2_ were determined using a binding energy difference set to a value of 1.2 eV and an intensity ratio of 2:1. Ar ion-beam etching (energy of 4 keV; emission current of 10 mA) was used to obtain depth profiles (3.5 × 3.5 mm raster size). Prolonged ion-beam etching (>10 min) can provoke preferential Hg removal [65], leading to surface alteration visible under scanning electron microscopy. To avoid any experimentally induced alterations/modifications, ion-beam etching was limited to 2 min.

#### 4.4.4. SEM-EDX

Scanning electron microscopy (SEM) and image analysis were performed using a variable-pressure scanning electron microscope (VP-SEM, Hitachi SU3500, Hitachi High-Tech Corporation, Tokyo, Japan) operated in low-vacuum mode (30 Pa) at an accelerating voltage of 10 kV. The instrument was equipped with dual energy-dispersive X-ray spectroscopy (dEDS) detectors (Bruker XFlash^®^ 6|60; Bruker Nano GmbH, Berlin, Germany), which allowed simultaneous multimodal imaging and spatially resolved elemental mapping.

#### 4.4.5. FTIR-ATR Spectroscopy

FTIR (Fourier-transform infrared spectroscopy) analysis was carried out to investigate the chemical composition of the materials and to evaluate possible changes in the paint layer before and after artificial aging, as well as following the application of protective coatings. Measurements were performed in attenuated total reflection (ATR) mode, which does not require sample preparation. Spectra were collected using a Nicolet Summit FT-IR spectrometer (Thermo Fisher Scientific, Waltham, MA, USA) equipped with an Everest™ Diamond ATR accessory. The acquisition parameters included 32 scans per sample, a spectral resolution of 8 cm^−1^, and a measurement range from 4000 to 600 cm^−1^, allowing for accurate identification of chemical changes and degradation mechanisms.

#### 4.4.6. XRF

Portable energy-dispersive X-ray fluorescence (EDXRF) spectroscopy was carried out to investigate the elemental composition of the real sample and the laboratory replicas. Measurements were collected by using a portable EDXRF spectrometer (HITACHI X-MET8000 Expert) equipped with an Rh anode X-ray tube and a Si-PIN detector (Hitachi High-Tech Corporation, Tokyo, Japan). The acquisition parameters were set at 45 kV, 40 μA, and a live time of 60 s.

#### 4.4.7. Spectrocolorimetry

Spectrocolorimetry was utilized to define color variations in the cinnabar egg yolk paint induced by artificial aging and application of micro- and nanostructured coatings. Measurements (carried out in triplicate) were performed using the 3nh NS810 spectrophotometer (Shenzhen ThreeNH Technology Co., Ltd., Shenzhen, China), within the CIELab color space. Chromatic differences were calculated according to ΔE* (Equation (5)):

(5) $$\Delta E*=\sqrt{\left({\left({L_{2}}^{*}-{L_{1}}^{*}\right)}^{2}+{\left({a_{2}}^{*}-{a_{1}}^{*}\right)}^{2}+{\left({b_{2}}^{*}-{b_{1}}^{*}\right)}^{2}\right)}$$

In the CIELab system, the ΔE* value is defined by the three parameters L, a*, and b* and can be interpreted as follows: ΔE* < 3 corresponds to color variations imperceptible to the naked eye; 3 < ΔE* < 5 indicates perceptible but acceptable changes; and ΔE* > 5 denotes significant alterations, implying a modification of the artwork’s surface appearance [66]. These values were used to evaluate the susceptibility of the pigment, applied in egg tempera, to aging, specifically to the darkening phenomenon, and to assess the applicability of micro- and nanostructured coatings for the conservation of cultural heritage.

#### 4.4.8. Contact Angle Measurement

Contact angle measurements were conducted using an OCA 15EC (DataPhysics Instruments GmbH, Filderstadt, Germany). This analysis was applied to investigate how wettability changes as a result of aging, considering that aging and binder degradation tend to reduce hydrophobicity. The measurements were repeated after the application of the protective coatings to assess their effectiveness in restoring an adequate level of hydrophobicity. Measurements were performed using the sessile drop method with deionized water as the probe liquid. Each film was tested in triplicate, and the average values were recorded to ensure reproducibility.

#### 4.4.9. Additional Characterization

All the adhesion forces, drilling resistance, permeability (P%) and treatment efficiency (ET%) before and after eight months from the first application were measured according to the experimental procedures, widely reported in our previous paper [35] and briefly summarized in the Supplementary Materials (Table S6) [67,68,69,70].

## 5. Conclusions

This work allowed us to study the mechanism of chemical–physical damage induced by aging in α-HgS samples prepared in the laboratory, using an egg-based protein binder. The oxidation of Hg^2+^ was mainly observed, forming secondary chemical compounds such as HgSO_4_, calomel/Hg_2_Cl_2_, and HgCl_2_ (and β-HgS, even though it is very difficult to distinguish it from the other chemical shifts, among alpha-cinnabar, chlorine-Hg-based compounds and oxidized Hg^2+^ as HgSO_4_), while metallic Hg^0^ was never observed (after aging and after the hybrid coating applications). The FTIR study demonstrated that the presence of Hg^2+^ altered the chemical nature of the protein binder, causing red shift and intensity band modification, especially those attributed to the amide’s functional groups. The crystalline form of anatase, functionalized with hydroxyl groups (OH) on the surface, ensured that these groups act as quenchers, neutralizing reactive species before they can electrochemically transform the mercuric ion (Hg^2+^) into elemental mercury (Hg^0^). Furthermore, the presence of the hybrid coating undoubtedly protected against further decomposition of the egg-based binder, as FTIR analysis showed no additional red-shift effects or alterations in the band signal intensities associated with amide groups.

At the same time, functionalized TiO_2_ Ps were uniformly dispersed within the FCP matrix, minimizing the risk of forming aggregated TiO_2_ clusters, which could cause unwanted whitening effects on the surface of the cinnabar samples (in agreement with the colorimetric data). The results were also excellent in terms of contact angle, surface area coverage, total pore volume, permeability and efficiency of the treatment for at least 8 months from the first application, although studies are still underway (on the same samples) to establish the performances over longer time intervals.

In light of these important results, a hybrid coating was also applied to the original cinnabar samples (RC). XPS analysis showed (after treatments based on a hybrid coating) a chemical shift in Hg compatible with that of alpha-cinnabar, where no signals attributable to oxidized Hg^2+^ and/or chlorine-based products were observed. There was no presence of metallic Hg^0^, a finding also supported by SEM micrographs. The latter did not even show TiO_2_ particle aggregates, according to the colorimetric analysis. The optical and colorimetric properties of natural cinnabar will be the subject of further analysis, carried out in the presence of traces of beeswax, oils, and plant resins, the natural binders found in prehistoric adhesives, applied to fix cinnabar on archaeological artworks. The performance of the hybrid coating on RC was satisfactory regarding contact angle, surface area, total pore volume, tensile strength, vapor permeability, and treatment efficiency. In particular, the latter two parameters are still being measured in the same sample (to determine their values over longer time intervals).

Furthermore, Poggio Spaccasasso (Tuscany, Italy) was selected because it possesses numerous prehistoric archaeological artifacts composed of natural cinnabar mixed with trace amounts of natural binders, such as beeswax, oils, and plant resins. Such original specimens (i.e., RC) could represent a groundbreaking frontier for conservation science, as they would provide crucial information for understanding the prehistoric technologies, practices, and traditions of ancient civilizations, which undoubtedly influenced human evolution.

## Figures and Tables

**Table 1:** Binding energies (BEs) and deconvoluted peaks (%) for Hg, S, Cl, Se of Ctrl, A Ctrl and RC samples.

Peak BE (eV)	Deconvoluted Peaks (%)				Chemical Compounds
	Hg	S	Cl	Se	
Ctrl (no aging)					
Hg 4f_7/2_ (100.2 eV) Hg 4f_5/2_ (104.5 eV)	(75%)				Hg^2+^ (α-HgS)
S 2p_3/2_ (162.0 eV)		(15%)			S^2−^ (α-HgS)
S 2p_3/2_ (161.5 eV)		(5%)			S^2−^ (Hg_3_S_2_Cl_2_) with Hg^2+^
Cl 2p_3/2_ (199.0 eV)			(5%)		Cl^1−^ (Hg_3_S_2_Cl_2_) with Hg^2+^
A Ctrl (after aging)					
Hg 4f_7/2_ (100.8 eV) Hg 4f_5/2_ (104.9 eV)	(65%)				Hg^2+^ (β-HgS; HgSO_4_; HgCl_2_)
S 2p_3/2_ (162.5 eV) O 1 s (532.3 eV)		(15%)			(S^6+^O_4_) ^2−^ (HgSO_4_) with Hg^2+^
					S-O bonding on (SO_4_)^2−^
Cl 2p_3/2_ (198.5 eV)			(10%)		Cl^1−^ (Hg_2_Cl_2_) with Hg^1+^
Cl 2p_3/2_ (199.0 eV)			(10%)		Cl^1−^ (HgCl_2_) with Hg^2+^
Original cinnabar RC sample from Poggio Spaccasasso					
Hg 4f_7/2_ (100.1 eV) Hg 4f_5/2_ (104.3 eV)	(80%)				Hg^2+^ (α-HgS)
S 2p_3/2_ (162.2 eV) S 2p_1/2_ (163.4 eV)		(15%)			(S) ^2−^ (α-HgS) with Hg^2+^
Se 3d_5/2_ (55.1 eV)				(5%)	Se (HgSe) with Hg^2+^

**Table 2:** Chromatic variation (ΔE*, ΔH, and ΔC) and water contact angle (WCA/θ) of the laboratory-prepared samples. ΔE* formula corresponds to the CIE 1976 color difference equation.

Sample Labels	Colorimetry	ΔE*	ΔH	ΔC	WCA (θ)
Ctrl	L* = 46.20 ± 0.20 a* = 54.20 ± 0.30 b* = 35.89 ± 0.22	-	-	-	98° ± 3°
A Ctrl	L* = 46.95 ± 0.21 a* = 52.33 ± 0.25 b* = 33.04 ± 0.31	3.49 ± 0.20	1.38 ± 0.30	3.12 ± 0.10	50° ± 3°
FCP	L* = 45.19 ± 0.24 a* = 53.09 ± 0.26 b* = 33.53 ± 0.22	1.98 ± 0.10	0.01 ± 0.25	−0.91 ± 0.10	106° ± 2.5°
FCP_TiO_2_	L* = 45.04 ± 0.03 a* = 50.28 ± 0.02 b* = 30.31 ± 0.12	3.13 ± 0.03	1.18 ± 0.10	−2.53 ± 0.02	116° ± 2.0

**Table 3:** Kremer pigment properties.

Product	SEM Image	Dimension of Particles	Code
Cinnabar No. 10620 (Kremer) Provenance: Hunan Province (China)	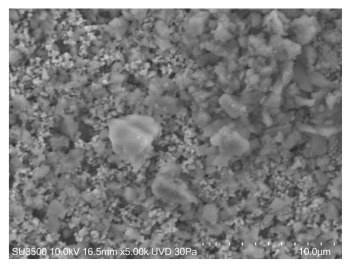	<20 μm	CIN-EF Cinnabar extra fine

**Table 4:** Textural features, water adsorption coefficient, permeability and CieLab test carried out on the original cinnabar natural stones (before and after the TiO_2_ Ps/FCP hybrid material treatments) and on aged laboratory samples (before and after the TiO_2_ Ps/FCP hybrid material treatments).

Samples	Surface Area (m^2^/g)	Total Pore Volume (cm^3^/g)	Adsorbent Amount of Products ^(1)^ (kg m^−2^)	Penetration Rate ^(2)^ (mm/min^0.5^)	IC_w_ (%)	WCA (θ)	P (%) Immediately After Applying and Drying the Products	P (%) After 8 Months from the First Application	Colorimetry
A Ctrl	2.0 ± 0.5	0.01 ± 0.3	--	--		50 ± 3°	--	--	L* = 46.95 ± 0.21 a* = 52.33 ± 0.25 b* = 33.04 ± 0.31
A TiO_2_Ps/FCP	0.5 ± 0.7	0.005 ± 0.5	1.1 ± 0.01	1.2 ± 0.05		90 ± 2°	7 ± 1.0	7 ± 1.0	L* = 43.24 ± 0.20 a* = 52.28 ± 0.22 b* = 32.41 ± 0.30
RC	8.0 ± 0.4	0.4 ± 0.2	--	--	100 ± 0.01	n.d. *	--	--	L* = 42.95 ± 0.10 a* = 22.91 ± 0.15 b* = 10.06 ± 0.12
RC TiO_2_Ps/FCP	1.2 ± 0.5	0.03 ± 0.4	2.4 ± 0.02	3.2 ± 0.12	1.62 ± 0.03	80 ± 3°	10 ± 1.5	10 ± 1.5	L* = 40.01 ± 0.05 a* = 23.41 ± 0.10 b* = 9.13 ± 0.12

^(1)^ The quantitative absorption of the consolidants was indirectly determined by dry weight measurements of the cylindrical samples (50 mm length; 15 mm diameter) before and after the treatment. The weight measurements were conducted after 6 weeks of storage at 23 C/60% RH, for full curing of the consolidants. After 6 weeks, the samples were dried in an oven at 40 C until a constant mass was achieved and, subsequently, equilibrated at room temperature for one hour. ^(2)^ The consolidants were applied to sandstone by capillary adsorption; the stone specimens were partially immersed (3 mm depth) in the liquid consolidating agent for 3 h, respectively. The application time was determined by measuring the evolution of the capillary fringe (mm) on the lateral surface of the specimens for 3 h. The latter was the time necessary for the wet fringe of the majority of the consolidants to reach the top of the specimens (3 cm). The space covered in the established time returned the penetration speed of the consolidating products. * The inability to determine a contact angle indicated that a water droplet was completely adsorbed to the surface of the material, and, therefore, the contact angle was approximately Ø.

**Table 5:** Characterization of untreated and treated original cinnabar rock samples in terms of cohesion forces and treatment efficiency.

Samples	Increment in Superficial Hardness	DR l = Drill Bit Ø 5 (mm)	Tensile Strength	ET (%) Immediately After Applying and Drying the Products	ET (%) After 8 Months from the First Application
	(%)	(N)	(MPa)	(%)	(%)
A Ctrl	--	--	10 ± 1.1	--	--
A TiO_2_Ps/FCP	30 ± 1.3	24 ± 1.2	22 ± 1.0	75 ± 1.2	75 ± 1.1
RC	--	--	12 ± 1.3	--	--
RC TiO_2_Ps/FCP	43 ± 1.5	32 ± 1.4	29 ± 1.5	84 ± 1.4	85 ± 1.3

**Table 6:** Description and name of the prepared samples.

Sample Names	Sample Labels	Description
Marble + reproduced cinnabar ^1^	Ctrl	Control is a marble sample where cinnabar has been placed
Aged (marble + reproduced cinnabar)	A Ctrl	Aged control
Marble + Fluoline CP	FCP	Marble support treated by the polymer Fluoline CP
Aged (Marble + Fluoline CP)	AFCP	Marble support treated by Fluoline CP and then aged according to Section 4.3
Marble + TiO_2_ Ps/FCP hybrid material	TiO_2_ Ps/FCP	Marble support treated by the hybrid materials
Aged (marble + TiO_2_ Ps/FCP) hybrid material	ATiO_2_ Ps/FCP	Marble support treated by the hybrid materials and then aged according to Section 4.3
Original cinnabar	RC	Real samples collected in Poggio Spaccasasso, as reported below

^1^ The painted laboratory samples with the red cinnabar pigment.

## Data Availability

The data will be made available upon request.

## Supplementary Materials

The following supporting information can be downloaded at https://www.mdpi.com/article/10.3390/molecules31142429/s1, Scheme S1: Synthesis route and characterization data of functionalized TiO2 particles; Scheme S2: Experimental results demonstrating the presence of OH scavengers and quenchers on TiO2 particles surfaces by FTIR technique; Table S1: Attribution of the main vibrational bands of the prepared laboratory samples before and after aging; Table S2: Attribution of the main vibrational bands of the prepared laboratory samples treated with FCP only before and after aging; Table S3: Attribution of the main vibrational bands of the prepared laboratory samples treated with hybrid TiO2 Ps/FCP coating before and after aging; Table S4: XRD analysis performed on the real cinnabar sample; Table S5: XRF quantitative analysis of the elements contained in the cinnabar original sample; Table S6: Mechanical test, adhesion forces, texture of the material, colorimetry measurements performed in this study and compared with the literature (summarized in this table for different cinnabar samples); Figure S1: FTIR spectrum of real cinnabar sample (wavelength region: 4000–800 cm−1); Figure S2: XRF spectroscopy of cinnabar real/original sample; Figure S3: EDX spectra of Ctrl and Actrl samples; Figure S4: EDX spectra of FCP sample; Figure S5: EDX spectra of TiO2 Ps/FCP sample; Figure S6: EDX of RC. References [1,35,46,48,50,53,54,55,56,57,62,63,67,68,69,70] were also cited in Supplementary Materials file.
