# Supplementary material for: Hybrid TiO2 Particles/Fluorinated Polymer as a Protective Layer for α-HgS Cinnabar: A Multi-Analytic Study
Source: Molecules. 2026 Jul 10;31(14):2429. doi: 10.3390/molecules31142429 (PMC13415997; doi:10.3390/molecules31142429)
Supplement: Supplementary file 1 [file molecules-31-02429-s001.zip › molecules-4300833-supplementary.pdf]

molecules

MDPI

Article

Hybrid TiO_2 Particles/Fluorinated Polymer as a Protective Layer for α-HgS Cinnabar: A Multi-Analytic Study

Federica Valentini [1,*], Pasquino Pallecchi [2], Irene Angela Colasanti [1,3], Camilla Zaratti [1,3], Andrea Macchia [3], Michela Relucenti [4], Loredana Cristiano [4], Nicoletta Volante [5], Ilaria Fratoddi [6] and Sara Cerra [6]

[1] Department of Sciences and Chemical Technologies, Tor Vergata University of Rome, Via della Ricerca Scientifica 1, 00133 Rome, Italy; ireneangela.colasanti@students.uniroma2.eu (I.A.C.); camilla.zaratti@students.uniroma2.eu (C.Z.)
[2] Museo e Istituto Fiorentino di Preistoria, Via dell'Oriolo 24, 50122 Firenze, Italy; pasquino.pallecchi@unifi.it
[3] YOCOCU APS, Via Torquato Tasso 108, 00185 Rome, Italy; aps@yococu.com
[4] Anatomical, Histological, Forensic and Orthopaedic Sciences Department, Sapienza University of Rome, Via Alfonso Borelli 50, 00161 Rome, Italy; michela.relucenti@uniroma1.it (M.R.); loredana.cristaino@uniroma1.it (L.C.)
[5] Historical Sciences and Cultural Heritage Department, Siena University, Via Roma 56, 53100 Siena, Italy; nicoletta.volante@unisi.it
[6] Department of Chemistry, Faculty of Science MFN, Sapienza University of Rome, P.le Aldo Moro 5, 00185 Rome, Italy; ilaria.fratoddi@uniroma1.it (I.F.); sara.cerra@uniroma1.it (S.C.)
* Correspondence: federica.valentini@uniroma2.it

Supplementary Materials

Characterization measurements (concerning Table 3 and Table 4 in main text).

Textural Properties. The specific surface area (Brunauer-Emmett-Teller, BET method) and total pore volume (by Gurvitsch (1915), [35]) were determined by adsorption/desorption of N_2 at -196 °C using a 3 Flex 3500 Micro metrics apparatus after sample outgassing at 200 °C for 2 h. The pore size distribution was determined by the Barrett-Joyner-Halenda (BJH) method [35], from the adsorption isotherm. The uncertainty for the values of specific surface area was ±0.5 $m^2 g^{-1}$.

Physical Properties. The physical characterization was performed bringing the sample (with dimension of 1.5x1.5x3 cm^3) to dryness at the working temperature of 60 °C, and then, the dry weight W_d was measured. The real volume V_r and the bulk volume V_b were quantified using a Quanta chrome helium pycnometer and a Chandler Engineer mercury pycnometer, respectively. Then the samples were dipped into deionized water and weighed after saturation (obtaining a constant wet weight W_w), [35]. By means of this data, it is possible to obtain IC_W, as reported below (Eq. 1):

water imbibition coefficient ICw%, (expressed in weight) = (Ww - Wd)/Wd x 100 (Eq. 1)

Water Vapor Permeability (P %). P% is quantified by vapor permeability measurements, using the formula (Eq. 2):

$$\boldsymbol{P}\,(\%) = \frac{(\boldsymbol{\theta_{NT}} - \boldsymbol{\theta_{T}})}{\boldsymbol{\theta_{NT}}} \times \mathbf{100} \qquad \textbf{(Eq. 2)}$$

where: θ_{NT} represents the steady state steam flow of sample without treatment based on TiO_2 Ps/FCP composites; θ_T represents the steady state steam flow of the treated sample with TiO_2 Ps/FCP; P (%) represents the decrease in vapor permeability following the application of the product [35 and references cited therein].

Surface hardness. Surface hardness was quantified with a Martens sclerometer, equipped with a two wheel handcart and a steel tip for scratching [35]. Measurements were carried out with the steel tip in contact with the specimen, ensuring it was perpendicular to the material surface. The upper surface of the handcart remained horizontal with the final force of 3 kgf applied on the tip. The handcart was subjected to a constant speed until reaching the set length. Every 3 mm along its length, the width of the incision was recorded by applying a movable magnifying glass equipped with a light source and a micrometer, with 0.02 mm as resolution. For each sample, 4–6 incisions were carried out, and the results as the width of stroke (WS) are reported as the average values.

Drilling Resistance Measurements. Test The micro-drilling resistance measurements were performed using the drilling resistance test (DRMS) [35 and references cited therein], mainly considered the most suitable method for the quantification of consolidation performances. For this investigation, 5 mm diameter tungsten drill bits have been applied, with a rotation speed of 400 rpm and penetration rate of 15 mm min^{-1}.

Tensile strength was determined by ASTM C297/C297M (2016), as reported in our previous paper [35 and references cited therein].

Treatment Efficiency (%). This property was quantified by applying the Equation (3), as reported below:

$$\boldsymbol{E_T}\,(\%) = \frac{\boldsymbol{M_{NT}} - \boldsymbol{M_T}}{\boldsymbol{M_{NT}}} \times \mathbf{100} \qquad \text{(Eq. 3)}$$

Scheme S1. Synthesis route and characterization data of functionalized TiO_2 particles.

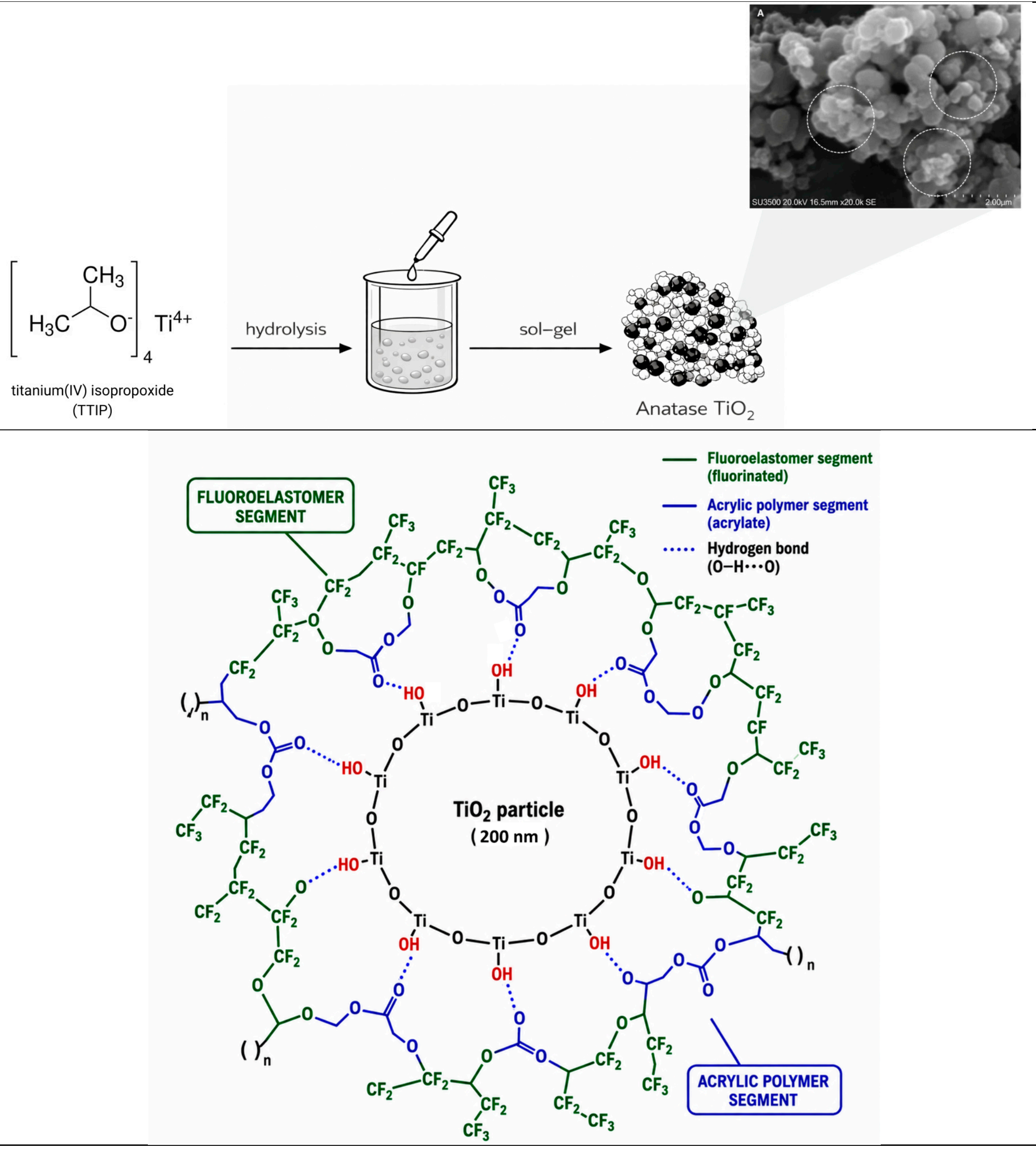

Characterization results of TiO_2 particles (Anatase phase)

Samples	SEM/EDX (nm)	FTIR (cm-1)	XRD Anatase	Z-potential (ξ, mV)	DLS (μm)	Acidic Sites(b) (nmol/mg)	Weight Loss (% TGA)	Surface Area (m2/g)	Pore Volume (cm3/g)	Pore Size (nm)
Pristine TiO_2 Ps	200 Spherical particles	420-550 δ Ti-O-Ti<br>1622 ν Ti-OH<br>3200-3600 ν OH	(a) (101) 25.3°<br>(004) 38.0°<br>(200) 48.1°<br>(105)/(211) 55.1°<br>(204) 62.7°	*--------	0.31	*--------	*--------	13.3	0.046	180

(a): Highest intensity, characteristic peak for Anatase; (b): These reactive functional groups were also quantified through the volumetric titration of the acidic sites, (mean ± SD) as reported in [35]

https://doi.org/10.3390/xxxxx

Scheme S2. Experimental results demonstrating the presence of OH scavengers and quenchers on TiO_2 particles surfaces, by FTIR technique**.**

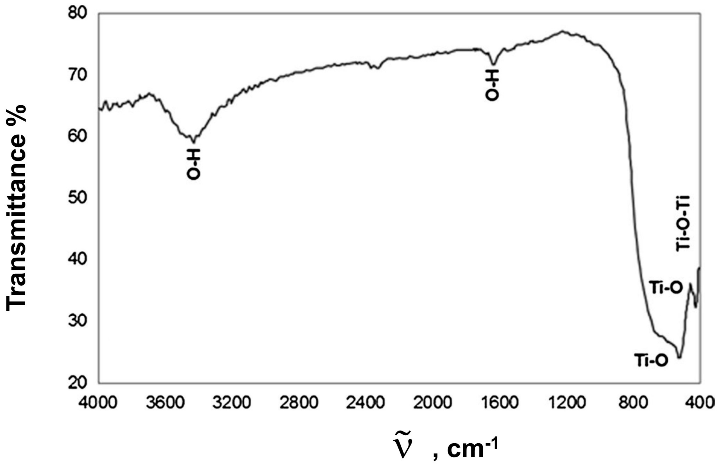

FTIR band assignment of TiO_2 NPs into FCP_TiO_2 hybrid samples		
Wavenumber, cm^{-1}	Normal Vibrational Mode	Functional groups
408.9	ν	Ti–O–Ti
447.0	ν	Ti-O
667.0	ν	Ti-O
1620	δ	OH
3450	ν	OH

https://doi.org/10.3390/xxxxx

Table S1. Attribution of the main vibrational bands of the prepared laboratory samples before and after the aging.

**Ctrl non-aged (cm^{-1})**	**ACtrl aged (cm^{-1})**	**Assignment**	**Notes**	**Reference**
3257	Attribution of the main vibrational bands of the prepared laboratory samples before and after the aging. 3251	νO–H (bound water)<br>N–H (proteins)	As a result of artificial aging, it decreases; there is a lower number of H-bonds, due to protein denaturation and lipidic oxidation	[1]<br>[48]<br>[49]<br>[59]
2950	2951	ν_{as}C–H (CH_3)	*	
2917	2917	ν_{as}C–H (CH_2)	*	
2870	2866	ν_{s}C–H (CH_3)	*	
2852	2848	ν_{s}C–H (CH_2)	*	
1739	1735	νC=O	Increased absorbance due to lipid oxidation and formation of new carbonyl compounds.	
1629	1623	νC=O (Amide I)<br>νCOO-	As a result of artificial aging, it decreases for denaturation	
1539	1525	δN–H<br>νC–N (Amide II)	Increase after aging process. This behavior is consistent with protein denaturation, as reported in the literature, where the Amide II/Amide I ratio increases due to conformational changes and the formation of β-sheet structures.	
1451	1452	δCH_2	Increased absorbance due to lipid degradation and accumulation of aliphatic oxidative products.	
1417	1401	δCH_2		
1368	1374	δCH_3		
1227	1220	νC–N (Amide III) + νP=O	Increase in absorbance but loss of definition, due to protein denaturation	
1151	1150	νC–O	Increased absorbance due to lipid hydrolysis and oxidative formation of alcohols, acids and esters.	
1066	1081	νC–O-C		
983	971	CH deformation	*	
844	859	δCH + CH deformation	*	
710	706	CH deformation	*	
	659	νC–C	New band formed, linked to secondary products	

*: The fingerprint of the carbon atom chain structure does not show significant changes after aging.

Table S2. Attribution of the main vibrational bands of the prepared laboratory samples treated with FCP only before and after aging.

**FCP non-aged (cm^{-1})**	**A FCP aged (cm^{-1})**	**Assignment**	**Notes**	**Reference**
3257	3251	νO–H (bound water) N–H (proteins)	Decrease after aging due to reduced hydrogen bonding and partial degradation of organic components	
2980	2981	ν_{as}C–H (CH_3)	Aliphatic chains of the polymer, no significant variation after aging	
2950	2951	ν_{as}C–H (CH_3)	Aliphatic chains of the polymer matrix	
2917	2917	ν_{as}C–H (CH_2)	Aliphatic backbone; stability indicates limited degradation	
2870	2866	ν_{s}C–H (CH_3)	Symmetric stretching of methyl groups	
1730	1730	νC=O	Ester groups, no significant increase, indicating limited oxidative processes compared to untreated samples	[53] [54] [55]
1629	1623	νC=O (Amide I) + νCOO-	Residual organic material from substrate	
1539	1525	δN–H + νC–N (Amide II)	Slight variation; less pronounced increase compared to Ctrl samples, indicating protective effect of FCP	
1450	1452	δCH_2	Bending of aliphatic chains	
1417	1401	δCH_2; $\nu sCOO^-$	Minor contribution of oxidation products	
1368	1374	δCH_3	Methyl bending vibrations, slight increase	
1230	1225	νC–F (CF_3)	Characteristic band of fluorinated polymer	
1151	1150	νC–F (CF_2)	Fluoropolymer backbone	

Table S3. Attribution of the main vibrational bands of the prepared laboratory samples treated with hybrid TiO_2 Ps/FCP coating before and after aging.

**TiO_2 Ps/FCP non-aged (cm^{-1})**	**A TiO_2 Ps/FCP aged (cm^{-1})**	**Assignment**	**Notes**	**Reference**
3260	3263	νO–H (bound water) N–H (proteins)	Broad band due to adsorbed water and hydroxyl groups on TiO_2 surface; reduced intensity compared to Ctrl indicates protective effect	
2980	2981	ν_{as}C–H (CH_3)	Aliphatic chains of the polymer	
2920	2919	ν_{as}C–H (CH_2)	Aliphatic backbone	
2850	2849	ν_{s}C–H (CH_2)	Symmetric CH_2 stretching	
1730	1735	νC=O	Ester group, weak increase; lower than Ctrl indicating reduced oxidation processes	
1630	1636	νC=O (Amide I) δH–O–H	Contribution from proteins residue from substrate; also influenced by TiO_2 surface hydroxyls	[53] [54] [55] [56] [57]
1530	1560	δN–H + νC–N (Amide II) + νCOO-	Shift toward higher wavenumbers suggests interaction between polymer matrix and TiO_2 nanoparticles	
1450	1458	δCH_2	Bending of aliphatic chains; stable intensity indicates limited degradation	
1230	1232	νC–F (CF_3)	Characteristic fluoropolymer band	
1150	1148	νC–F (CF_2)	Fluoropolymer backbone	
1050	1055	νC–O-C	Polymer matrix	

https://doi.org/10.3390/xxxxx

800-600	800-600	ν + δ (Ti–O–Ti)	Broad band due to TiO_2 lattice vibrations; confirms nanoparticle presence and stability after aging

Table S4. XRD analysis performed on the real cinnabar sample

Sample ID	Qz	Ca	HgS	He	Gh	Ph
SPS#6 – F. Exterior. Encasing rock	Tr	xx	x			
SPS#6 – F. Exterior. Red Pigment	Tr	Tr	xx			
SPS#6 – Stone tools for cinnabar extraction	x			xx	x	

Table legend: Qz = Quartz; Ca = Calcite; HgS = Cinnabar; He = Hematite; Gh= Ghoetite; Ph= Phyllosilicates; Tr= traces; x = relative abundance

XRD apparatus and measurement procedures. X-ray diffraction (XRD) using Cu K_α radiation operating at 40 keV and 20 mA in θ-2θ X'Pert PRO Phillips diffractometer (20°–60° 2θ range) was used to characterize the structure and crystallinity of cinnabar original stones. Table below, shows the characteristic XRD fingerprint of α-HgS.

2θ Angle (degrees)	d-spacing (Å)	Relative Intensity (I / I_0)	Miller Indices (h; k; l)
26.6°	3.35	100% (Major Peak)	(0;1;1)
28.2°	3.16	65%	(1;0;2)
30.9°	2.89	45%	(1;1;0)
43.7°	2.07	45%	(1;0;4)
45.9°	1.97	30%	(0;2;2)
51.8°	1.76	35%	(1;1;4)

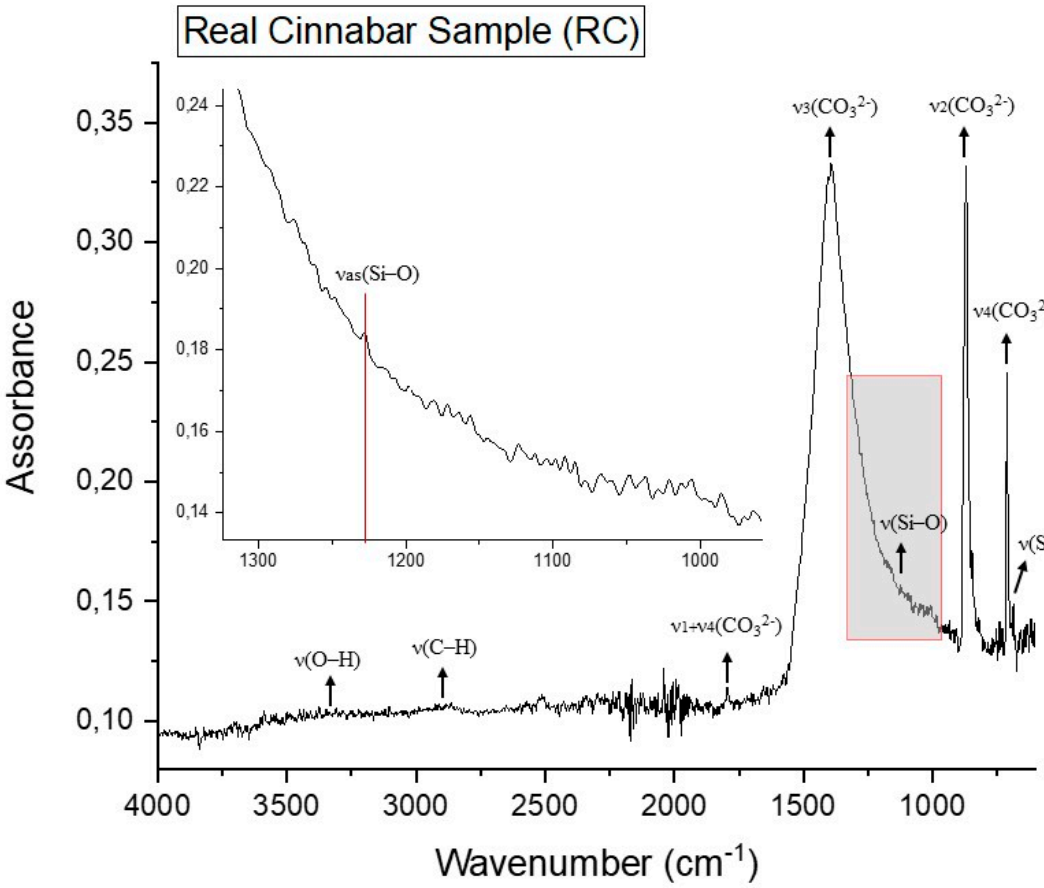

Figure S1. FTIR spectrum of real cinnabar sample, wavelength region 4000-800 cm^{-1}.

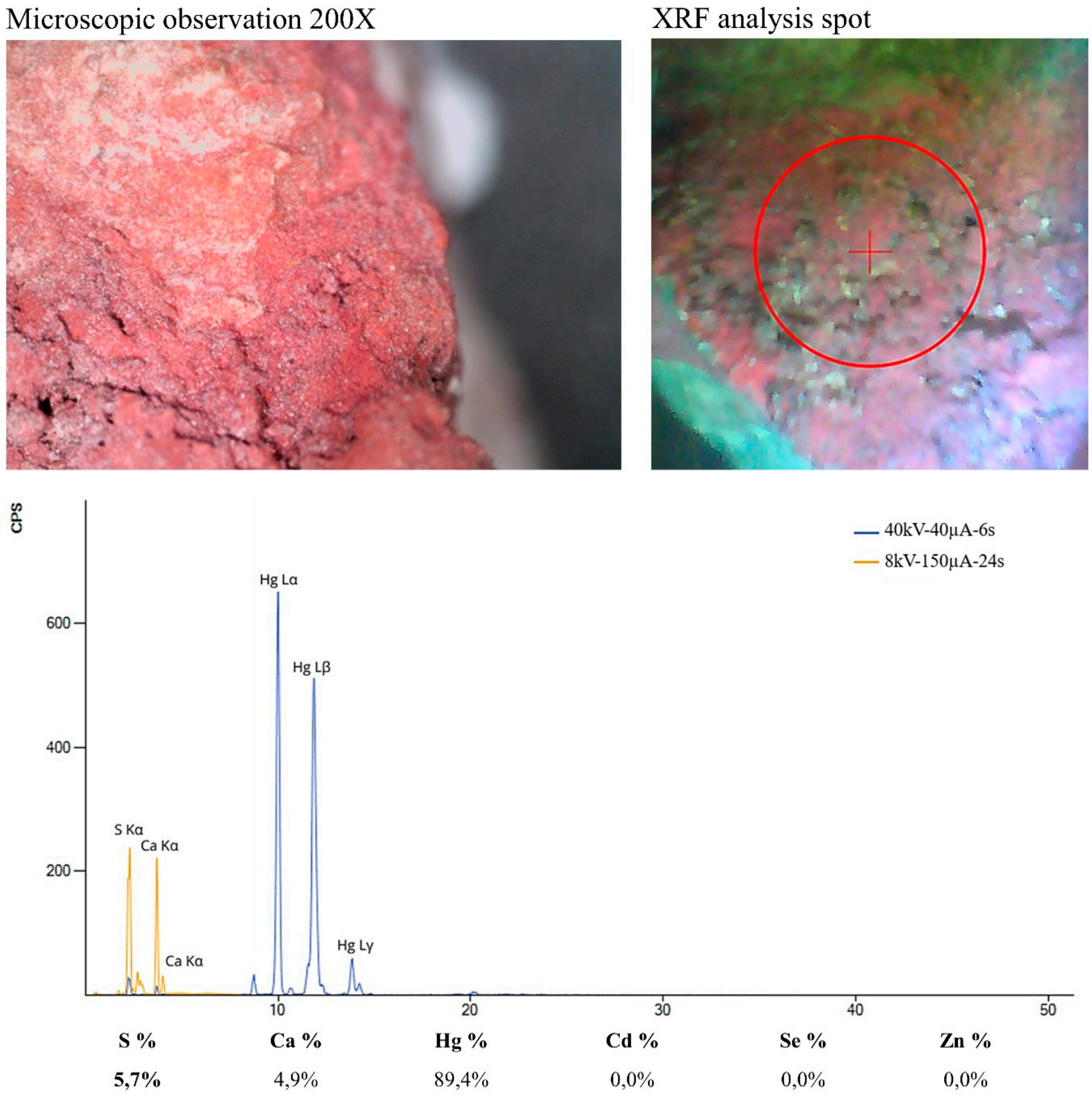

Figure S2. XRF spectroscopy of Cinnabar Real/Original Sample

Table S5. XRF quantitative analysis of the elements contained in the Cinnabar original sample

Element	%	Atomic weight	Moles	Total moles	Atomic %
S	5,70	32,00	0,18		23,88
Ca	4,90	40,08	0,12	0,75	16,39
Hg	89,40	200,60	0,45		59,74

Table S6. Mechanical test, adhesion forces, texture of the material, colorimetry measurements performed in this study and compared with literature (summarized in this Table for different cinnabar samples)

**Mechanical test**	**[67]**	**[68]**	**[69]**	**[70]**	**This work**
Cinnabar samples:	wood/mortar support	Chinese ancient paintings	thermally modified spruce wood	ancient wooden architecture in China	Original rock/stone (Tuscany, Italy)
Cinnabar conservative treatments:	SiO_2/TiO_2 + fluoropolymer	Water-Borne Fluoropolymer	Nano-cerium dioxide + polyurethane	FEVE (fluorocarbon resin solution) AC33 (acrylic resin primal)	TiO_2 Ps/FCP
Surface Area* (m2/g)					X
Total pore Volume* (cm3/g)					X
Adsorbent amount of products (kg m-2)					X
Penetration Rate (mm/min0.5)					X
ICw (%)					X
WCA (θ)	X	X	X	X	X
P (%) Immediately after applying and drying the products	X			X	X
P (%) after 8 Months from the first application					X
Colorimetry	X	X		X	X
Increment of Superficial Hardness (%)	X		X	X	X
Drilling Resistance DR (N)		X	X		X
Tensile Strength (MPa)	X	X	X	X	X
ET (%) immediately after applying and drying the products					X
ET (%) after 8 months from the first application					X

*: These quantities expressing the texture refer to the measurement on the original cinnabar sample (before and after the treatments); but they do not concern at all the nanoparticles/inorganic filler inside the polymer composite.

EDX analysis and related spectra

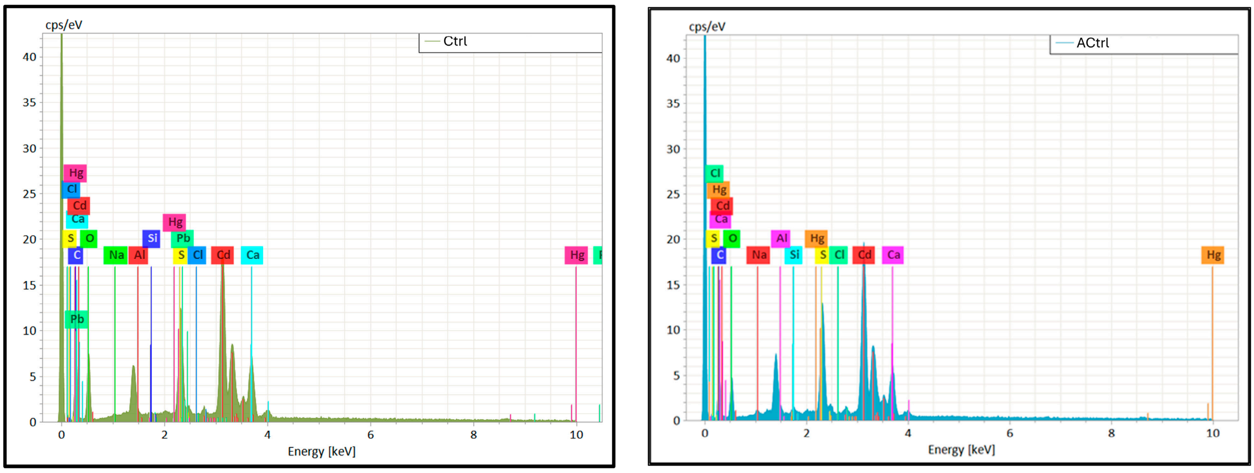

Figure S3. The EDX spectrum of Ctrl reveals the major component (Hg and S) assigned to α-HgS, and the minor components as Ca, Si, Al, Na (from the mock-up support). The presence of Pb and Cd could be present (as trace geological impurities) in the sample of Natural Cinnabar (Red Cinnabar, often mined in China), produced by Kremer Pigmente (code 10620). Cl elements in Ctrl could be also related to the $Hg_3S_2Cl_2$ (corderoite, a rare mercury sulfochloride mineral), present as trace geological impurities. However, for chlorine species, an origin linked to the photodegradation processes of cinnabar cannot be ruled out, as is also evident from the microanalysis spectrum shown alongside for A Ctrl [62].

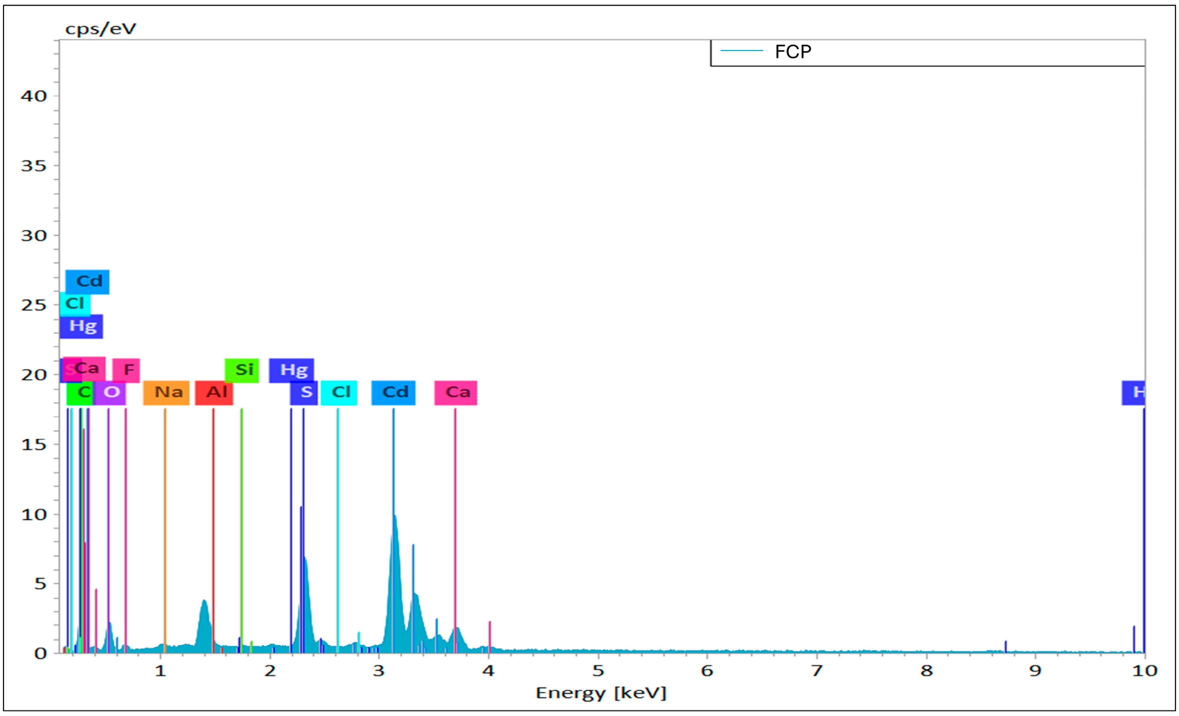

Figure S4. The EDX spectrum of FCP sample reveals the presence of Hg, S, Ca, Si, Al, Cl, Cd. The difference with the EDX spectrum recorded for Ctrl sample is in the presence of the F element (attributable to the application of the FCP polymer-based treatment).

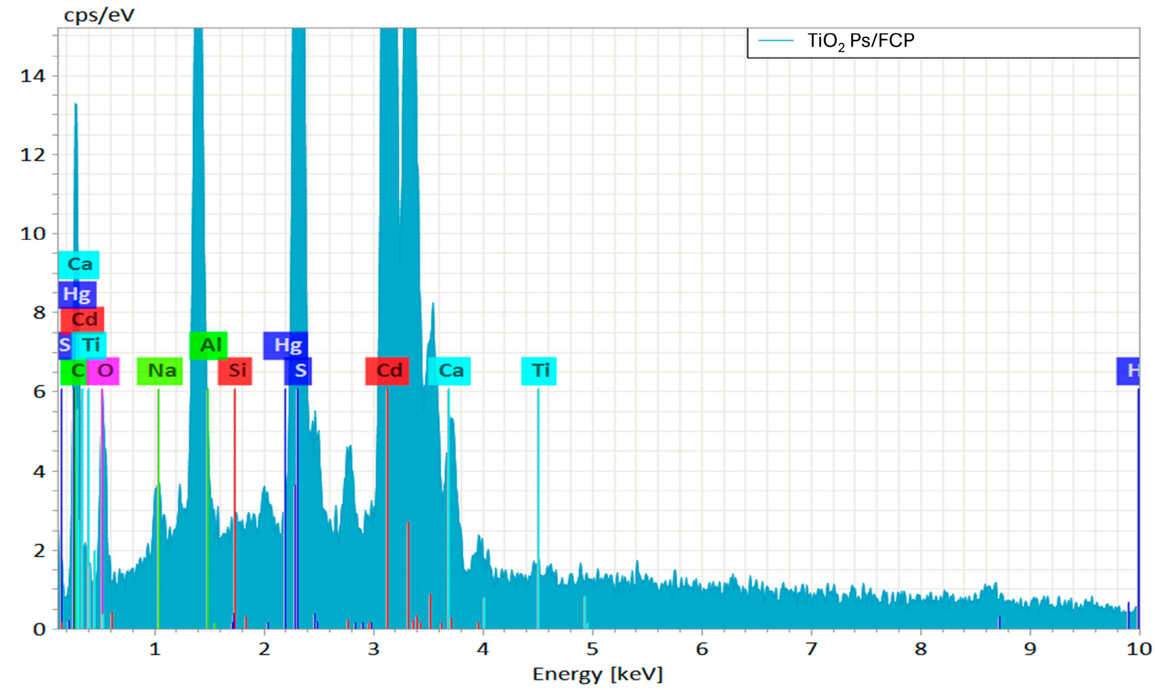

Figure S5. The EDX profile shows the presence of Hg, S, Si, Al, Ca, Cd, Cl, O and Ti elements (especially this latter for the entrapment of TiO_2 particles into the hybrid materials).

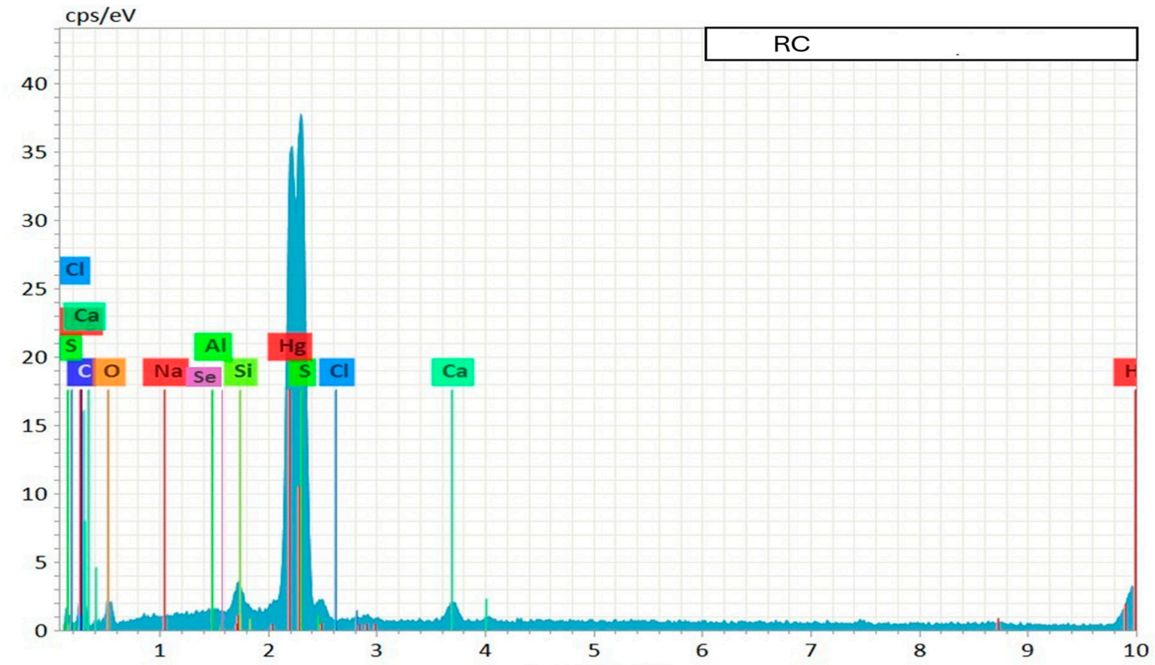

Figure S6. The EDX spectrum shows the majority components, which are attributable to Hg and S (α-HgS in RC). Minor components are also observed, such as Si, Ca, Al, Fe (this last as He-Hematite, also confirmed by XRD analysis, reported on Table S4) and Se element. This latter/Se, can replace sulfur in the crystal lattice of cinnabar, forming natural solid solutions of mercury-selenium sulfide (Hg (S, Se)). The presence of chlorine/Cl can be traced back to the chemical-physical and photochemical decomposition processes, to which natural cinnabar (α-HgS) is subject in the environment [46].

References on Supporting Information also reported in the main text final Bibliography

1. Elert, K.; Cardell, C. Weathering Behavior of Cinnabar-Based Tempera Paints upon Natural and Accelerated Aging. Spectrochimica Acta Part A Molecular and Biomolecular Spectroscopy 2019, 216, 236–248. https://doi.org/10.1016/j.saa.2019.03.027.

35. Valentini, F.; Pallecchi, P.; Relucenti, M.; Donfrancesco, O.; Sottili, G.; Pettiti, I.; Mussi, V.; De Angelis, S.; Scatigno, C.; Festa, G. SiO_2 Nanoparticles as New Repairing Treatments toward the Pietraforte Sandstone in Florence Renaissance Buildings. Crystals 2022, 12 (9), 1182. https://doi.org/10.3390/cryst12091182

46. Botticelli, M. *Archaeometric Investigations on Red Pigments: The Provenance of Cinnabar and the Discrimination of Synthetic and Natural Ochres*; Ph.D. Thesis, Sapienza Università di Roma, Rome, Italy, 2016. Available online: https://iris.uniroma1.it/retrieve/e3835322-16ad-15e8-e053-a505fe0a3de9/Tesi_dottorato_Botticelli.pdf (accessed June 26, 2026).

48. Mazzeo, R.; Prati, S.; Quaranta, M.; Joseph, E.; Kendix, E.; Galeotti, M. Attenuated Total Reflection Micro-FTIR Characterisation of Pigment–Binder Interaction in Reconstructed Paint Films. Analytical and Bioanalytical Chemistry, **2008**, 392, 65–76. https://doi.org/10.1007/s00216-008-2126-5.

53. Cai, X.; Lei, T.; Sund, D. and Lin, L. A critical analysis of the α, β and γ phases in poly(vinylidene fluoride) using FTIR. *Journal RSC Advance*, Issue 25, **2017.** https://doi.org/10.1039/C7RA01267E

54. Toniolo, L.; Poli, T.; Castelvetro, V.; Manariti, A.; Chiantore, O.; and Lazzari, M. Tailoring new fluorinated acrylic copolymers as protective coatings for marble. *Journal of Cultural Heritage* 3(4):309-316, **2002.** https://doi.org/10.1016/S1296-2074(02)01240-2.

55. Al-Amin, Kazi and Kawsar, Md. and Mamun, Md. Tariqur Rahaman Bhuiyan and Sahadat Hossain, Md. Fourier transform infrared spectroscopic technique for analysis of inorganic materials: a review. *Nanoscale Adv.*, Vol. 7, No.21, pp.6677-6702. **2025.** DOI: https://doi.org/10.1039/D5NA00522A

56. Valentini, F.; Colasanti, I. A.; Zaratti, C.; Filimon, D.; Macchia, A.; Neri, A.; Michela Relucenti; Massimo Reverberi; Allegrini, I.; Guerriero, E.; Cerasa, M.; Luca, M. D.; Santangeli, F.; Braglia, R.; Scuderi, F.; Rugnini, L.; Ranaldi, R.; Meis, R. D.; Canini, A. TiO_2 and $CaCO_3$ Microparticles Produced in Aqueous Extracts from Satureja Montana: Synthesis, Characterization, and Preliminary Antimicrobial Test. *Molecules* **2025**, *30* (20), 4138–4138**.** https://doi.org/10.3390/molecules30204138.

57. Han, K.; Ge, M.; Li, K. Conservation of color paintings on the Rain Flower Pavilion using a multi-functional composite coating. *npj Herit. Sci.* **2025**, *13*, 266. https://doi.org/10.1038/s40494-025-01812-w

62. Kremer Pigmente GmbH & Co. KG. *Cinnabar, 10620: Product Information Sheet*; Kremer Pigmente: Aichstetten, Germany. https://www.kremer-pigmente.com/elements/resources/products/files/10620e.pdf (accessed June 26, 2026).

63. Socrates, G. Infrared and Raman characteristic group frequencies: tables and charts, 3rd ed. John Wiley & Sons Ltd., New York (USA). **2004**

50. Silverstein, R. M.; Webster, F. X.; Kiemle, D. J. Spectrometric Identification of Organic Compounds, 7th ed.; Wiley: New York, **2005**.

67. Scarfato, P.; Fariello, M.L.; Di Maio, L.; Incarnato, L. Weatherability Evaluation of Nanocomposite Polymeric Treatments for Surface Protection of Construction Materials. *AIP Conf. Proc.* 1255, 373–375 **2010.** https://doi.org/10.1063/1.3455640

68. Wang, J.; Hu, D.; Xing, H.; Qi, Y.; Li, Y. Facile and Scalable Conservation of Chinese Ancient Paintings Using Water-Borne Fluoropolymer. *ACS Omega* **2020**, *5* (51), 33162-33169. https://doi.org/10.1021/acsomega.0c04827

69. Mastouri Mansourabad, A.; Azadfallah, M.; Tarmian, A. Nano-cerium dioxide synergistic potential on abrasion resistance and surface properties of polyurethane-nanocomposite coatings for esthetic and decorative applications on wood. *J Coat Technol Res.* **2020**, *17*, 1559–1570. https://doi.org/10.1007/s11998-020-00374-9

70. Han, K.; Teri, G.; Cheng, C. Evaluation of commonly used reinforcement materials for color paintings on ancient wooden architecture in China. *Herit. Sci.* **2024**, *12*, 122. https://doi.org/10.1186/s40494-024-01236-y
